# Pancancer transcriptomic profiling identifies key PANoptosis markers as therapeutic targets for oncology

**DOI:** 10.1093/narcan/zcac033

**Published:** 2022-11-01

**Authors:** Raghvendra Mall, Ratnakar R Bynigeri, Rajendra Karki, R K Subbarao Malireddi, Bhesh Raj Sharma, Thirumala-Devi Kanneganti

**Affiliations:** Department of Immunology, St. Jude Children's Research Hospital, Memphis, TN 38105, USA; Department of Immunology, St. Jude Children's Research Hospital, Memphis, TN 38105, USA; Department of Immunology, St. Jude Children's Research Hospital, Memphis, TN 38105, USA; Department of Immunology, St. Jude Children's Research Hospital, Memphis, TN 38105, USA; Department of Immunology, St. Jude Children's Research Hospital, Memphis, TN 38105, USA; Department of Immunology, St. Jude Children's Research Hospital, Memphis, TN 38105, USA

## Abstract

Resistance to programmed cell death (PCD) is a hallmark of cancer. While some PCD components are prognostic in cancer, the roles of many molecules can be masked by redundancies and crosstalks between PCD pathways, impeding the development of targeted therapeutics. Recent studies characterizing these redundancies have identified PANoptosis, a unique innate immune-mediated inflammatory PCD pathway that integrates components from other PCD pathways. Here, we designed a systematic computational framework to determine the pancancer clinical significance of PANoptosis and identify targetable biomarkers. We found that high expression of PANoptosis genes was detrimental in low grade glioma (LGG) and kidney renal cell carcinoma (KIRC). *ZBP1, ADAR, CASP2, CASP3, CASP4, CASP8* and *GSDMD* expression consistently had negative effects on prognosis in LGG across multiple survival models, while *AIM2, CASP3, CASP4* and *TNFRSF10* expression had negative effects for KIRC. Conversely, high expression of PANoptosis genes was beneficial in skin cutaneous melanoma (SKCM), with *ZBP1, NLRP1, CASP8* and *GSDMD* expression consistently having positive prognostic effects. As a therapeutic proof-of-concept, we treated melanoma cells with combination therapy that activates ZBP1 and showed that this treatment induced PANoptosis. Overall, through our systematic framework, we identified and validated key innate immune biomarkers from PANoptosis which can be targeted to improve patient outcomes in cancers.

## Graphical Abstract

**Graphical Abstract:**
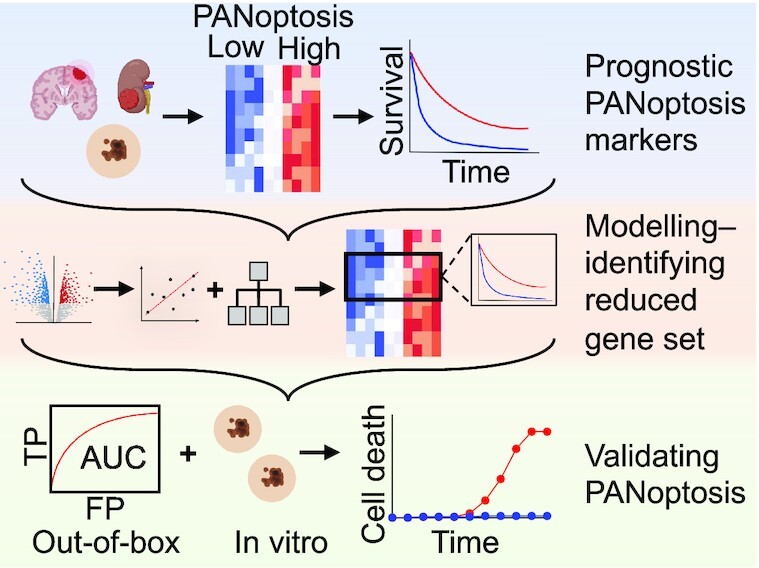
PANoptosis (27-gene set) clusters are prognostic pancancer; in silico framework identifies reduced PANoptosis gene sets which are validated to correlate with clinical prognosis and drive cell death (PANoptosis) in vitro.

## INTRODUCTION

Resistance to cell death is one of the hallmarks of cancer (1). Activation of programmed cell death (PCD) pathways can be a successful strategy to clear cancer cells (2,3). Several distinct innate immune-mediated PCD pathways have been identified (4), with pyroptosis, apoptosis and necroptosis being the best-characterized. Understanding the clinical impact of PCD in cancer prognosis is important for patient stratification and identifying the molecular mechanisms of cancer pathogenesis, making this an active area of research (5–13). However, much remains unknown. Furthermore, the roles of many PCD molecules can be masked by functional redundancies, synergisms and crosstalks between PCD pathways. These redundancies have made it difficult to identify specific molecular targets in PCD for anti-cancer therapies. Therefore, studies that consider the totality of biological effects in PCD to identify molecular functions and therapeutic targets are critical.

Recent studies highlighting the extensive crosstalk among the molecular components of pyroptosis, apoptosis and necroptosis in infectious diseases, autoinflammatory diseases and cancers have led to the identification of PANoptosis, a unique innate immune-mediated inflammatory PCD pathway regulated by PANoptosome complexes, which integrate components from other PCD pathways (14–27). To date, PANoptosis has been functionally assessed most extensively *in vitro* and in murine models. In a murine model of colorectal cancer, IRF1 drives PANoptosis to inhibit tumorigenesis (28). Additionally, treatment with IFN combined with a nuclear export inhibitor activates PANoptosis and limits tumorigenesis in murine models of melanoma (17). In human cancer cell lines, PANoptosis can be activated by TNF-α and IFN-γ co-treatment to kill the cancer cells (18). Despite these advances, little is known about the prognostic implications of PANoptosis for overall survival (OS) in human patients with diverse cancers. While the roles of pyroptosis, apoptosis and necroptosis independently in pancancer progression and therapeutic responses have been investigated (6,29), considering these pathways as segregated entities and not as an integrated PCD modality through PANoptosis provides an incomplete understanding of PCD in cancer.

In this study, we developed a systematic framework using 32 tumor lineages from The Cancer Genome Atlas (TCGA) (30) to characterize the prognostic implications of PANoptosis gene expression. We defined the PANoptosis gene signature based on experimental evidence from the literature (16,17,31–33) and used a consensus clustering approach (34–39) to classify tumor samples into PANoptosis high, PANoptosis medium and PANoptosis low groups for each cancer type. We then devised a two-step computational approach to filter the gene set and determine the key PANoptosis molecules of consistent clinical relevance for OS, and we validated these models using independent (out-of-box) test sets.

We found that high expression of PANoptosis genes was detrimental for OS in low grade glioma (LGG) and kidney renal cell carcinoma (KIRC), while it was beneficial in skin cutaneous melanoma (SKCM). The expression of *ZBP1, ADAR, CASP2, CASP3, CASP4, CASP8* and *GSDMD* consistently contributed to the negative effect on prognosis in LGG, while *AIM2, CASP3, CASP4* and *TNFRSF10* consistently contributed in KIRC. In SKCM, *ZBP1, NLRP1, CASP8* and *GSDMD* expression consistently contributed to the positive effect on prognosis. As a proof-of-concept that the biomarkers we identified could be translated into potential therapeutic targets, we treated melanoma cells with combination therapy that activates ZBP1 (17) and showed that this treatment induced PANoptosis to kill the cancer cells. Overall, our findings underscore the importance of PANoptosis for the prediction of cancer patient survival and suggest new biomarkers which can be targeted to improve patient outcomes.

## MATERIALS AND METHODS

### Data acquisition, filtering and normalization

RNA-Seq data from TCGA (https://www.cancer.gov/tcga) were downloaded and processed using TCGA biolinks (v2.22.3). The RNA-Seq data from 32 primary solid tumor (TP) cancers consisting of over 9000 tumor samples were used in our analysis. Owing to the lack of TP samples in SKCM, we included metastatic samples (TM) in the SKCM dataset. Gene symbols were converted to the official HUGO Gene Nomenclature Committee gene symbols, and genes without gene symbols or gene information were excluded. This resulted in 23 216 genes for each cancer type. For each cancer type, the samples were quantile normalized using preprocessCore (v1.56.0) and log_2_ transformed for further analysis.

To compare expression of the genes of interest in a tissue specific manner, quantile normalized RNA-Seq data were obtained from the UCSC Xenabrowser (https://toil.xenahubs.net). The RNA-Seq dataset comprised a comprehensive set of tumor samples from TCGA as well as healthy controls from Genotype-Tissue Expression Project (GTEx) (40) for different tissues of origin. This dataset contained a total of 19 120 samples, 10 535 samples from TCGA and 7781 samples from GTEx, and was used for differential expression analysis. Moreover, phenotypic information, such as the age, gender, grade/stage for each cancer patient in TCGA was obtained from the UCSC Xenabrowser.

### Out-of-box validation datasets

For the out-of-box validation of the prognostic value achieved by the survival models built for various cancer subtypes, independent test sets were obtained from the PREdiction of Clinical Outcomes from Genomic profiles (PRECOG) repository (41) as well as the National Center for Biotechnology Information (NCBI) GEO Accession viewer. Gene expression profiles of patients from GSE22155 (42) and GSE16011 (43) were obtained, along with their survival information from PRECOG repository. Expression profiles of patients from GSE65904 (44) were acquired through GEO Accession viewer and E-MTAB-1980 (45) through the ArrayExpress along with their corresponding survival information. For each of these external datasets, only those samples with survival information available were considered. For the datasets obtained through PRECOG repository, the ‘getGEO’ function from GEOquery package (v2.62.2) was used to acquire the gene expression profiles and download the raw counts for the other datasets. Expression profiles of genes with missing gene symbols were then removed. Each external dataset was then further quantile normalized, and log_2_ transformation was performed to utilize the processed data for testing the predictive capabilities of the survival models.

GSE65904 and GSE22155 were used as validation sets for SKCM survival models and consisted of 202 and 54 samples, respectively. GSE16011 consisted of 284 samples, of which only 20 samples belonged to Grade II Astrocytes or Grade II Oligodendrocytes, i.e., the LGG tumor subtype; these 20 samples were treated as the validation set for LGG. Finally, E-MTAB-1980 (NG2699), consisting of 101 samples, formed the test set for KIRC.

### Cancer cell lines

A total of 1377 cancer cell lines along with their expression profiles were downloaded from DepMap portal (DepMap Public 21Q3). These cell lines belong to the Cancer Cell Line Encyclopedia (46). The cell lines were filtered to include only those cell lines for which the primary disease associated was skin cancer, resulting in 34 melanoma cancer cell lines. These cancer cell lines had inherent diversity in terms of the age, gender, cancer type (primary or metastasis) of the skin cancer patients as well as their sample collection site.

We performed experimental validation on two melanoma cancer cell lines: SK-MEL-5 (NCI-60 cancer cells; National Cancer Institute, Bethesda, MD) and RVH-421 (DSMZ) cancer cells. SK-MEL-5 was derived from the lymph node of a 24-year-old female and is a metastatic cancer cell line. The RVH-421 cell line was derived from the central nervous system of a 28-year-old male and is a metastatic cancer cell line.

### Single cell transcriptomics

Single cell transcriptomics datasets for LGG and SKCM cancer types were downloaded from the Broad Institute Single Cell Portal under accession number SCP271 (47) and GEO Accession viewer under accession ID GSE72056 (48), respectively.

The metadata for the tumor of origin and cluster labels for the single cells in the LGG scRNA-seq dataset were also available. The LGG dataset comprised single cell transcriptomics of six different Pilocytic Astrocytomas (PA) consisting of a total of 931 cells. The Seurat (v4.1.1) package in R (49) with default normalization steps was used to process the dataset. These steps include normalizing using ‘LogNormalize’ method with a scale factor of 10 000 followed by selection of the top 3000 genes with maximal variance using the ‘vst’ method and scaling the dataset. Principal Component Analysis (PCA) (50) was then performed, with the number of principal components set to the default setting of 30. Then the Unified Manifold Approximation and Projection (UMAP) (51) method was run, resulting in the 2D co-ordinates for the single cells and allowing visualization of the LGG dataset.

The SKCM single cell RNA-seq (scRNA-seq) consisted of single cells derived from six patients each with at least 50 malignant cells as well as their corresponding non-malignant (immune and endothelial) cells. The dataset consisted of a total of 3700 cells. The same pre-processing steps as those outlined for the LGG dataset were used to obtain the UMAP 2-D co-ordinates for the SKCM dataset.

### PANoptosis clusters

**Figure 1.**
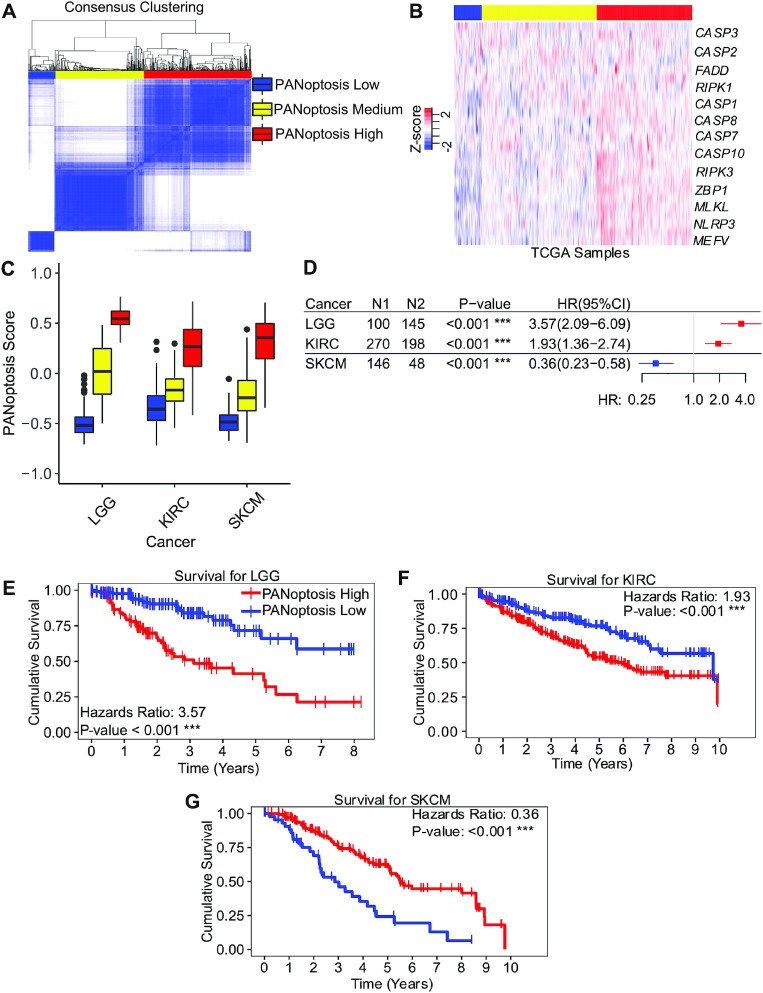
PANoptosis has a prognostic impact in cancers. (**A**) Consensus Clustering showing three distinct clusters (PANoptosis low, PANoptosis medium and PANoptosis high) based on PANoptosis gene expression for SKCM. (**B**) Heatmap depicting gene expression profiles of 27 PANoptosis markers including sensors and upstream regulators, adaptors and effectors of PANoptosis as scaled Z-scores for SKCM tumor samples. For brevity, 13 out of the 27 genes are labeled, but 27 distinct rows are shown. (**C**) Boxplot showing the distribution of PANoptosis scores in the three PANoptosis clusters for cancer subtypes of interest: LGG, KIRC and SKCM. (**D**) Forest plot showing N1 = number of samples in PANoptosis high cluster, N2 = number of samples in PANoptosis low cluster, *P*-value and hazard ratio (HR) with 95% CI for overall survival (OS) when comparing PANoptosis high versus low for each cancer type where there is significant prognostic impact (*P*-value < 0.05). (**E–G**) Kaplan–Meier curves showing OS across the PANoptosis high and PANoptosis low groups in the three cancer types with significant differences in survival (PANoptosis high beneficial [HR < 1] or detrimental [HR > 1]). *** *P*-value < 0.001.

An unsupervised consensus clustering based on a gene set of 27 PANoptosis genes (Supplementary Table S1) was performed for each cancer type separately using the ConsensusClusterPlus (v.1.58.0) R package with the following parameters: 5000 repeats, a maximum of six clusters and agglomerative hierarchical clustering with Ward criterion (‘ward.D2’) inner and complete outer linkage. This methodology has previously been shown to be successful in identifying optimal prognostic clusters for pancancer immunologic constant of rejection (52–55) and pyroptosis-related signatures in gastric cancer (56). The optimal number of clusters (≥3) for best segregation of samples based on the PANoptosis signature was initially determined heuristically using the Calinski-Harabasz criterion (57). With the intent to compare cancer samples with a highly active PANoptosis phenotype with those that have a relatively inactive PANoptosis phenotype, the cluster with the highest average expression of PANoptosis genes was designated as ‘PANoptosis high’, while the cluster with the lowest average expression of PANoptosis genes was designated ‘PANoptosis low’. All samples in the intermediate cluster(s) were defined as ‘PANoptosis medium’. Tumor samples were annotated with a PANoptosis score, defined as the single sample gene set enrichment score (ssGSEA) obtained from the GSVA (v1.42.0) R package (58) using the ‘gsva’ function with the kernel density parameter set as ‘Gaussian’. For generation of the Heatmap (Figure 1B), a modified version of ‘heatmap.3’ function was used.

### Survival analysis

Overall survival (OS) from the TCGA clinical data resource was used to generate the Kaplan–Meier (59) curves. For each cancer type, patients with less than one day of follow-up were removed, and survival data were censored after a follow-up duration of 10 years. The hazard ratios (HRs) between PANoptosis high and PANoptosis low clusters, including their corresponding *P*-values, were estimated through a χ^2^ (chi-square) test (60). The ‘analyze_survival’ followed by ‘kaplan_meier_plot’ functions from survivalAnalysis (v0.2.0) R package were used to build the univariate survival analysis models and visualize the Kaplan–Meier plots, respectively. The forest plots (Figure 1D, Supplementary Figure S2B) were generated using the forestplot (v2.0.1) R package. The cancer types, pheochromocytoma and paraganglioma (PCPG, no death events in PANoptosis high), kidney chromophobe (KICH, one death event in PANoptosis high and four death events in PANoptosis low), testicular germ cell tumors (TGCT, no death events in PANoptosis low) and pancreatic adenocarcinoma (PAAD, no death events in PANoptosis low) were excluded before the generation of the forest plot, as the number of deaths in the two comparison groups (PANoptosis high versus PANoptosis low) was too small for survival estimation. Cancer types with a *P*-value <0.05 and a total number of tumor samples >100 in PANoptosis high plus low groups were identified as cancers where PANoptosis had a significant prognostic value.

Three types of survival analysis models were built with increasing order of complexity to filter and extract the subsets of key PANoptosis genes driving the survival prognosis in the cancer subtypes of interest. The simplest model was the univariate cox-proportional hazards model (61), performed using the ‘coxph’ function from the survival (v3.2.11) R package. A multivariate cox-proportional hazards model (Coxnet) was also built, taking into consideration all the relevant PANoptosis markers using the ‘coxph’ function. To test the proportional hazards (PH) assumption, the ‘cox.zph’ function was utilized. This function correlated an individual PANoptosis marker's scaled Schoenfeld residuals (61) with time in order to test for independence between the residuals and time.

A regularized cox elastic-net regression (62,63) model using the glmnet (v4.1.3) package in R was then used. The regularized cox regression model is a generalized linear model (GLMnet) with an additional regularization term on hazards coefficients (β) to be estimated for the PANoptosis genes. The regularization path for either the Least Absolute Shrinkage and Selection Operator (LASSO) (62) or elastic-net model was obtained through a grid search using cross-validation. The optimal regularization parameter (λ) value was identified using the ‘glmnet.cv’ function.

Finally, the non-linear random-forest survival model (64) was used with the ‘rfsrc’ function from the randomForestSRC (v3.0.0) R package. The hyper-parameter optimization for parameters, such as ‘mtry’ or number of genes used to build a tree, ‘ntree’ or number of trees, and ‘nodesize’ or the size of nodes of a tree, was performed using a grid-search approach with cross-validation. The models were built by randomly sampling 80% of the data as a training set, with the remaining used as the out-of-box validation set. The PANoptosis genes which were important in the optimal random-forest model were identified using the fast ‘subsample’ function, which prioritized the most important genes along with a confidence interval for their importance.

The predictive capability of a survival model was measured through quantitative metrics, referred to as Harrell's concordance index (CI) (65), and the area under the time-dependent receiver operating curve (AUC) (66). The CI was defined as the proportion of concordant pairs divided by the total number of possible evaluation pairs. It ranged between [0,1], where values closer to 1 indicated that the predicted risk scores were almost completely correlated with survival, and CI values >0.5 were considered better than random predictions. The AUC metric determined the predictive capability of a model at time (*t*). In particular, the AUC metric would be higher if a model could accurately determine the at-risk patients at a time point (*t*) (true positives) with fewer false positives, that is, patients who are alive at time point (*t*) but had higher risk scores from the model. The AUC metric takes values between [0, 1], where higher AUC values (closer to 1) indicated better predictive performance of the model, and AUC values >0.5 were considered better than random predictions.

### Differential expression analysis

**Figure 2.**
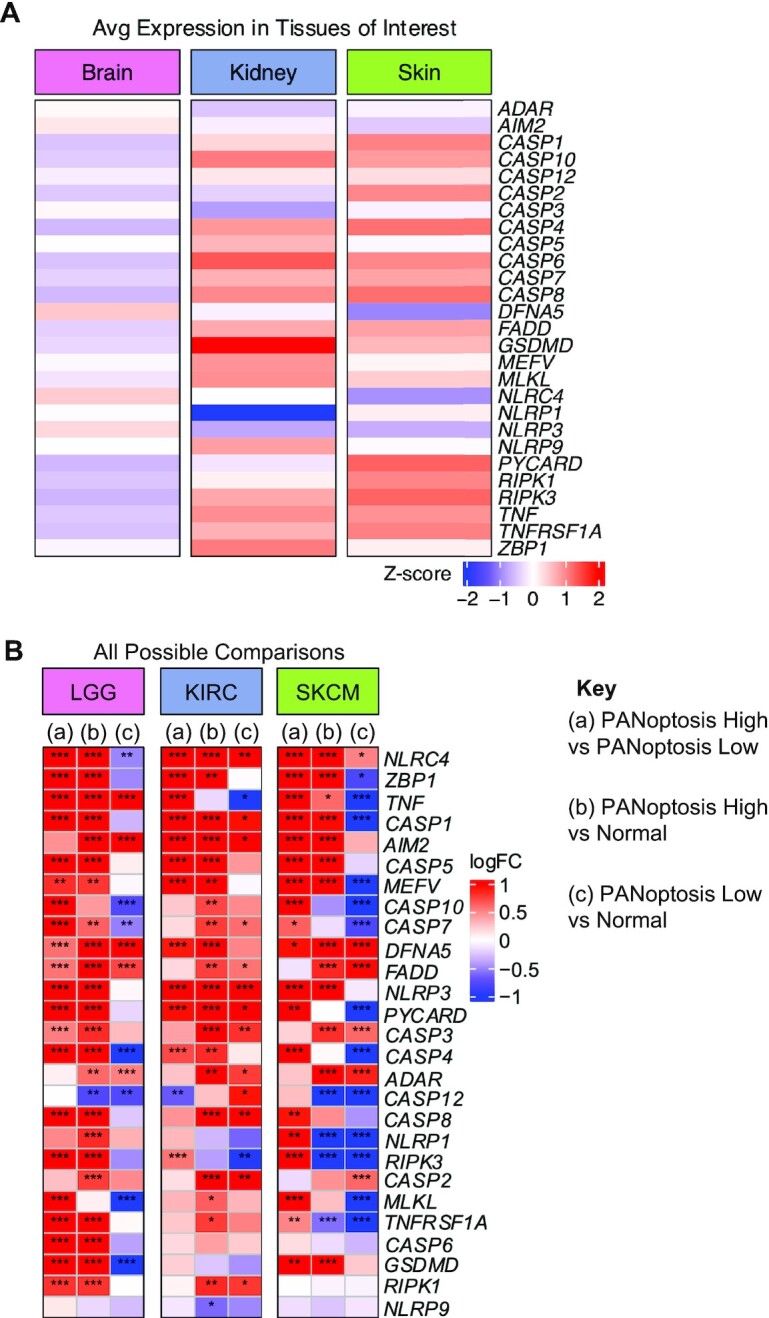
PANoptosis markers are differentially expressed across tissues and tumor types. (**A**) Basal expression of the 27 PANoptosis markers across the three normal tissue types of interest. (**B**) LogFC (log_2_ fold-change) matrix illustrating the differential regulation of the PANoptosis markers for (a) PANoptosis high versus PANoptosis low; (b) PANoptosis high versus normal; (c) PANoptosis low versus normal for the three cancer types. A gene is considered differentially expressed if |logFC| > 0.5 and FDR-adjusted *P*-value < 0.05. *Gene with an FDR-adjusted significance of differential expression with a *P*-value ∈ (1e–5, 1e–2]; **P-value ∈ (1e–10, 1e–5]; ***P-value < 1e–10.

To identify gene sets which were differentially regulated between two conditions (e.g. PANoptosis high vs PANoptosis low, PANoptosis high versus normal and PANoptosis low versus normal, see Figure 2B), the Limma (v3.50.0) R package (67) was used. The ‘model.matrix’ function was used to estimate the design matrix, followed by the ‘lmFit’ and ‘eBayes’ functions to determine the linear fitted model. The ‘topTable’ function was employed to obtain the results from the linear fitted model with statistical information. Only those genes with a false discovery rate (FDR) (68) adjusted *P*-value <0.01 and |log_2_(fold change)| >0.5 were considered as differentially expressed genes. Here, |^.^| corresponds to absolute value function.

### Gene set enrichment analysis

To define the PANoptosis score (Figure 1C, Supplementary Figures S2A, S8), a single sample gene set enrichment analysis (ssGSEA) was performed using the ‘gsva’ method with kernel density function parameter set to Gaussian kernel. To estimate the PANoptosis activity (enrichment) for each cell in the single cell transcriptomics dataset, we used the ‘enrichIt’ function from escape (v1.6.0) package in R (69). The ‘enrichIt’ function implements ssGSEA specific to single cell transcriptomic data.

### Correlation matrix

The correlation matrix of all PANoptosis genes was calculated using Pearson correlation and plotted using ComplexHeatmap (v2.10.0) R package (70). The significance of correlations between the expression profiles of two genes was estimated using the ‘cor.test’ function with the method parameter set as Pearson correlation.

### Cell culture and stimulation

RVH-421 (ACC 127) was obtained from DSMZ-German Collection of Microorganisms and Cell Cultures, and SK-MEL-5 (HTB-70) was obtained from ATCC as part of the NCI-60 cancer cell line panel. Each cell line was cultured in RPMI 1640 medium (Corning, 10–040-CV) supplemented with 10% FBS and 1% penicillin and streptomycin. Cancer cells were seeded at a concentration of 3 × 10^5^ cells into 12-well plates and incubated at 37°C overnight. Cells were washed with warm Dulbecco's PBS before being treated with 10 μM KPT-335 (Selleckchem, S7707) or 5 ng/ml leptomycin B (LMB; Sigma, L2913) in the presence or absence of 50 ng/ml IFN-γ (PeproTech, 300–02) in RPMI 1640 medium supplemented with 10% FBS and 1% penicillin and streptomycin for 32 h.

### Real-time imaging for cell death

The kinetics of cell death were determined using the IncuCyte S3 (Sartorius) live-cell imaging system. Cancer cells (3 × 10^5^ cells/well) were seeded in 12-well tissue culture plates. Cells were treated with the indicated stimuli and stained with propidium iodide (PI; Life Technologies, P3566) following the manufacturer's protocol. The plate was scanned, and fluorescent and phase-contrast images (4 image fields/well) were acquired in real-time every 1 h from 0 to 32 h post-treatment. PI-positive dead cells were marked with a ‘red’ mask for visualization. The image analysis, masking, and quantification of dead cells were done using the software package supplied with the IncuCyte imager.

### Western blotting

Western blotting in cancer cell lines was performed as previously described (18). Briefly, RVH-421 cells were seeded a day before stimulation at a density of 0.5 × 10^5^ cells/well in 6-well cell culture plates. For caspase evaluation, the proteins were collected by combining cell lysates with culture supernatants in caspase lysis buffer (with 1× protease inhibitors, 1× phosphatase inhibitors, 10% NP-40, and 25 mM DTT) and 4× sample loading buffer (containing SDS and 2-mercaptoethanol). For all other signaling proteins, the cells were lysed in RIPA buffer, supplemented with protease inhibitor and phosphoStop as per the manufacturer's instructions and sample loading buffer. Samples were denatured by boiling for 10 min at 100°C and separated using SDS-PAGE—followed by the transfer on to Amersham Hybond P polyvinylidene difluoride membranes (10600023; GE Healthcare Life Sciences) and immunoblotted with primary antibodies against caspase-1 (ab207802; Abcam), caspase-3 (9662; Cell Signaling Technology), cleaved caspase-3 (9661; Cell Signaling Technology), caspase-7 (9492; Cell Signaling Technology), gasdermin D (96458; Cell Signaling Technology), GSDME/DFNA5 (ab215191; Abcam), caspase-8 (clone 12F5, ALX-804-242-C100; Enzo Life Sciences), RIPK3/RIP3 (NBP2-24588; Novus Biologicals) and β-actin (clone 13E5, 4970; Cell Signaling Technology) followed by secondary anti-rabbit or anti-mouse HRP antibodies (Jackson ImmunoResearch Laboratories).

### RT-PCR analysis

Total RNA was extracted using TRIzol (ThermoFisher Scientific, 15596026), and cDNA was prepared using 500 ng of total RNA using the High-Capacity cDNA Reverse Transcription Kit (Applied Biosystems, 4368814). Real-time quantitative PCR was then performed on an Applied Biosystems 7500 real-time PCR instrument with 2× SYBR Green (Applied Biosystems, 4368706). Primer sequences used were: *ZBP1* forward: 5′-AACATGCAGCTACAATTCCAGA-3′; *ZBP1* reverse: 5′-AGTCTCGGTTCACATCTTTTGC-3′; *β-ACTIN* forward: 5′-CACCATTGGCAATGAGCGGTTC-3′; *β-ACTIN* reverse: 5′-AGGTCTTTGCGGATGTCCACGT-3′.

### Statistical analysis

GraphPad Prism version 8.0 software was used for data analyses. Data were shown as mean ± SEM. Statistical significance was determined by two-way ANOVA (with Dunnett or Tukey multiple comparisons tests) for three or more groups. The numbers of experimental repeats and technical replicates are indicated in the corresponding figure legends; *n* = the number of biological replicates used in the experiments. * *P* < 0.05 and is considered statistically significant.

## RESULTS

### Prognostic impact of PANoptosis clusters in different cancer subtypes

To improve our understanding of the role of cell death in cancer and to determine whether PANoptosis has prognostic value in this context, we evaluated the expression of PANoptosis markers across cancer types. RNA-Seq data from 32 different cancer types (Table 1) were obtained from TCGA. We used experimental evidence in the PANoptosis literature (16,17,31–33) combined with known molecules from pyroptosis, apoptosis or necroptosis to identify a set of molecules involved in PANoptosis (Supplementary Table S1). The PANoptosis gene signature was defined as 27 genes including cytosolic sensors and upstream regulators (*ADAR*, *AIM2, MEFV, NLRC4, NLRP1, NLRP3, NLRP9, TNFRSF1A, ZBP1*), adaptors (*FADD, PYCARD*) and effectors (*CASP1, CASP10, CASP12, CASP2, CASP3, CASP4, CASP5, CASP6, CASP7, CASP8, DFNA5, GSDMD, MLKL, RIPK1, RIPK3, TNF*) (Supplementary Table S1) (33,71,72). To group tumor samples based on the gene expression profiles of PANoptosis markers, we performed an unsupervised consensus clustering for each cancer type separately (SKCM provided as an example; Figure 1A). The consensus clustering approach identified three clusters referred to as ‘PANoptosis high’, ‘PANoptosis medium’ and ‘PANoptosis low’, where tumors belonging to the PANoptosis high cluster had a majority of the PANoptosis markers highly expressed, thereby suggesting the possibility of enhanced cell death through PCD, and vice versa for the PANoptosis low cluster.

**Table 1.** TCGA cancer abbreviations. Cancers of interest are highlighted in colors

Cancer	Cancer type	Full name
**LGG**	Primary	Brain Low Grade Glioma
**KIRC**	Primary	Kidney Renal Cell Carcinoma
**SKCM**	Metastatic	Skin Cutaneous Melanoma
STAD	Primary	Stomach Adenocarcinoma
COAD	Primary	Colon Adenocarcinoma
ACC	Primary	Adrenocortical Carcinoma
BLCA	Primary	Bladder Urothelial Cancer
BRCA	Primary	Breast Invasive Carcinoma
CESC	Primary	Cervical Squamous Cell Carcinoma and Endocervical Adenocarcinoma
CHOL	Primary	Cholangiocarcinoma
ESCA	Primary	Esophageal Carcinoma
GBM	Primary	Glioblastoma Multiforme
HNSC	Primary	Head and Neck Squamous Cell Carcinoma
KICH	Primary	Kidney Chromophobe
PAAD	Primary	Pancreatic Adenocarcinoma
THYM	Primary	Thymoma
KIRP	Primary	Kidney Renal Papillary Cell Carcinoma
LIHC	Primary	Liver Hepatocellular Carcinoma
LUAD	Primary	Lung Adenocarcinoma
DLBC	Primary	Lymphoid Neoplasm Diffuse Large B-cell Lymphoma
MESO	Primary	Mesothelioma
OV	Primary	Ovarian Cystadenocarcinoma
PCPG	Primary	Phenochromocytoma & Paraganglioma
PRAD	Primary	Prostate Adenocarcinoma
READ	Primary	Rectum Adenocarcinoma
TGCT	Primary	Testicular Germ Cell Tumors
THCA	Primary	Thyroid Carcinoma
UCS	Primary	Uterine Carcinosarcoma
UVM	Primary	Uveal Melanoma
UCEC	Primary	Uterine Corpus Endometrial Carcinoma
LUSC SARC	Primary Primary	Lung Squamous Cell Carcinoma Sarcoma

We observed that the expression of PANoptosis genes varied between the tumor samples, reflective of intratumor heterogeneity (Figure 1B). Furthermore, several PANoptosis genes had correlated expression profiles across all tumor samples within a particular tumor type (Supplementary Figure S1A–C). Next, we estimated a PANoptosis score for a given tumor sample using a ssGSEA technique. The PANoptosis score was quantified as the difference between the average expression of PANoptosis markers versus the average expression of other genes (∼23 000 genes) in a tumor sample (73). Thus, a high PANoptosis score represented an overexpression of PANoptosis genes (or an enhanced possibility of cell death), while a low PANoptosis score represented down-regulation of the PANoptosis genes in a given tumor sample (or a reduced possibility of cell death). In line with this definition, we observed that PANoptosis scores were lower in tumors belonging to the PANoptosis low cluster when compared to the PANoptosis medium cluster, and the PANoptosis scores were lower in samples of the PANoptosis medium cluster in comparison to the PANoptosis high cluster (Supplementary Figure S2A).

The distribution of PANoptosis scores varied among the tumor samples, and the difference between the highest and lowest PANoptosis scores varied between the different cancer subtypes (Supplementary Figure S2A). We noticed a stark contrast between the median PANoptosis scores in PANoptosis high and PANoptosis low groups respectively for each of the LGG, KIRC and SKCM cancer types (Figure 1C). We therefore sought to investigate the more clinically relevant questions such as how the presence of two contrasting PANoptosis clusters (PANoptosis high versus PANoptosis low) contributed to survival and how this varied across diverse cancer subtypes.

To determine the clinical relevance of the PANoptosis clusters, we performed a univariate survival analysis for each of the 32 different cancer subtypes comparing the survival of patients with tumors in the PANoptosis high cluster (treatment group) to that of patients with tumors in the PANoptosis low cluster (control group). The quantitative difference in survival was measured via hazard ratio (HR) along with a 95% confidence interval (denoted in parentheses). An HR above a value of 1 suggested that patients with tumors in the PANoptosis low cluster had a better survival prognosis than those with tumors in the PANoptosis high cluster; an HR below a value of 1 suggested patients with tumors in the PANoptosis high cluster had a better survival prognosis than those with tumors in the PANoptosis low cluster.

The PANoptosis high phenotype was associated with significantly reduced OS (*P*-value < 0.001) in LGG and KIRC (Figure 1D and Supplementary Figure S2B). In LGG, we observed the greatest detrimental and significant association between the expression of PANoptosis genes and prognosis, with the largest HR of 3.57 (2.09–6.09) and the widest gap between the survival curves of patients in the PANoptosis low versus PANoptosis high clusters (Figure 1D, E). Similarly, for KIRC, PANoptosis high had a significant negative prognostic impact, with an HR of 1.93 (1.36–2.74) (Figure 1D, F). In contrast, the PANoptosis high cluster showed a significant survival benefit (*P*-value < 0.001) compared with the PANoptosis low cluster for the SKCM cancer type (Figure 1D and Supplementary Figure S2B). We observed a significant prognostic benefit for PANoptosis gene expression in SKCM, with an HR of 0.36 (0.23–0.58) and a large gap between the survival curves of patients in the PANoptosis high versus PANoptosis low clusters (Figure 1D, G). We also observed a significant prognostic association of PANoptosis with OS for cancers such as uveal melanoma (UVM), adrenocortical carcinoma (ACC), thymoma (THYM) and mesothelioma (MESO). However, owing to the small sample size (N1 + N2 < 100), we did not consider these cancer subtypes further in our analysis. Together, these results suggested that patients could be clustered into three different groups within each tumor type: PANoptosis high, PANoptosis medium and PANoptosis low with respect to the gene expression profiles of PANoptosis markers. Moreover, the PANoptosis high and PANoptosis low clusters were associated with OS trends for three cancer subtypes, highlighting their clinical relevance and the importance of personalized therapeutic regimens.

We built additional multivariate Coxnet survival models including patient clinical features such as age, gender, grade or stage of cancer in combination with the PANoptosis score for each of the three selected cancer subtypes. We observed that the PANoptosis score built using TCGA data was significantly prognostic in the multivariate Coxnet model for LGG, with an HR of 4.48 (2.56–7.84), and for KIRC, with an HR of 1.99 (1.29–3.06) (Supplementary Figures S3A, B); in both these cancer types, a higher PANoptosis score was significantly detrimental for OS. Conversely, we observed that a higher PANoptosis score was significantly beneficial in the multivariate Coxnet model for SKCM, with an HR of 0.291 (0.169–0.502) (Supplementary Figure S3C). These results illustrate that the PANoptosis score holds significance in OS prediction as a factor independent of confounding variables such as age, gender and molecular subtype of the tumor.

### Tissue and tumor-specific expression of PANoptosis markers

Different tissues express different basal levels of PANoptosis genes (74). This suggests that the underlying tissue type may pre-dispose certain cancers to have more of a PANoptosis high or PANoptosis low phenotype. To understand how the PANoptosis gene cluster expression in tumors differs from its expression in normal tissue, we characterized the tissue-specific expression of the 27 genes considered in the PANoptosis gene set based on the tissues of origin for the three tumor subtypes where PANoptosis clusters had a significant prognostic value (LGG, KIRC and SKCM). The average expression of these genes in normal samples for the three different tissues (brain, kidney and skin) was collected from GTEx (Figure 2A). The expression values of these genes were scaled across the tissues thereby demonstrating that the majority of the PANoptosis genes had higher basal expression in skin and kidney tissues when compared to their expression in the brain; notable exceptions were *NLRC4* and *NLRP3* sensors and the effector gene *DFNA5* (*GSDME*). Additionally, we observed tissue-specific lower basal expression of certain sensors such as *AIM2, NLRP3* and *NLRC4* in skin and *NLRP1* and *NLRP3* in kidney. We also observed lower basal expression of the effector gene *DFNA5* in skin in comparison to brain.

Our primary goal was to identify components of PANoptosis that could be targeted for therapeutic benefit. Therefore, we sought to reduce and filter the PANoptosis gene set from the original 27 genes to identify the minimal set of key targetable genes with relevance to survival for each of the three cancer subtypes of interest. To this end, we first compared the differential regulation of the PANoptosis markers for the following three conditions: (a) PANoptosis high vs PANoptosis low; (b) PANoptosis high versus normal; (c) PANoptosis low versus normal. We performed a differential expression analysis on the normalized RNA-Seq data downloaded from UCSC Xenabrowser comprising tumor and normal samples. The tumor samples from UCSC Xenabrowser were matched with those in the TCGA to distinguish the tumors into PANoptosis high and PANoptosis low clusters. We identified the differential expression of PANoptosis genes among all genes across the three comparison conditions (a, b and c) for each of the three cancer types (Supplementary Figures S4A–C). We also visualized the average expression of the 27 PANoptosis genes in normal versus PANoptosis low versus PANoptosis high samples for the three cancer types (Supplementary Figures S5A–C). We observed that in LGG and SKCM, the majority of PANoptosis genes had the highest expression in PANoptosis high cancer samples, the next highest expression in normal tissue, and the lowest expression in PANoptosis low cancer samples (Supplementary Figures S5A, C, D). In contrast, in KIRC, the majority of PANoptosis genes had the highest expression in PANoptosis high cancer samples followed by PANoptosis low cancer samples, and the lowest expression in normal tissue (Supplementary Figures S5B, D). This suggests that the majority of PANoptosis genes will be significantly upregulated when either comparing PANoptosis high versus PANoptosis low (a) or PANoptosis high versus normal (b) irrespective of the cancer subtype. Indeed, this is what we observed (Figure 2B). We also found that the majority of PANoptosis markers were significantly downregulated in LGG and SKCM when comparing PANoptosis low versus normal (c), while they were significantly upregulated in KIRC cancer subtype (Figure 2B).

For each cancer type of interest, the genes that were differentially regulated in condition (a) were referred to as the primary markers (Supplementary Table S2). Since these genes were significantly differentially regulated (|logFC| > 0.5 and FDR-adjusted *P*-value < 0.05) between PANoptosis high versus PANoptosis low clusters, and the patients in these clusters had significant differences in OS, we hypothesized that these genes are likely to play a prominent role in differential prognosis. PANoptosis genes which were significantly differentially regulated in conditions (b) or (c) were considered as secondary markers (Supplementary Table S2). Since these genes were differentially expressed when comparing the PANoptosis high/low cluster tumor samples to normal samples from GTEx, we hypothesized that they might also play an indirect role in the differential prognosis.

Based on this identification of primary and secondary markers that are likely most important for differential prognosis, we filtered our PANoptosis gene set for each cancer to remove genes which were not differentially regulated in any of the comparison conditions (a, b and c). This filtered out *NLRP9* for LGG and SKCM; *CASP6* for KIRC and SKCM; *RIPK1* for SKCM; and *GSDMD* and *NLRP1* for KIRC (Figure 2B and Supplementary Table S2). Together, these results indicate that tissue-specific basal expression of PANoptosis markers varies across the three healthy tissues of interest. While certain PANoptosis markers were consistently differentially regulated across the three cancer types, there were certain genes which were not differentially expressed for each of the cancer types. These genes can be eliminated to select for potentially targetable PANoptosis genes with clinical relevance.

### Identification of key prognostic PANoptosis markers through consensus of diverse survival analysis models

To further measure the predictive capability of the filtered PANoptosis markers for OS, we built diverse survival analysis models (62,64) of increasing complexity. The predictive capability of a survival model was quantitatively measured through Harrell's concordance index (CI) and area under receiver operating curve (AUC). We built survival models using only the primary markers as well as primary plus secondary markers for LGG, KIRC and SKCM cancer subtypes. We used a univariate, multivariate (Coxnet), generalized linear cox-regression analysis model (GLMnet) and a non-linear random-forest based survival (RFS) model for each of the three cancers using the primary or primary plus secondary markers as covariates.

**Figure 3.**
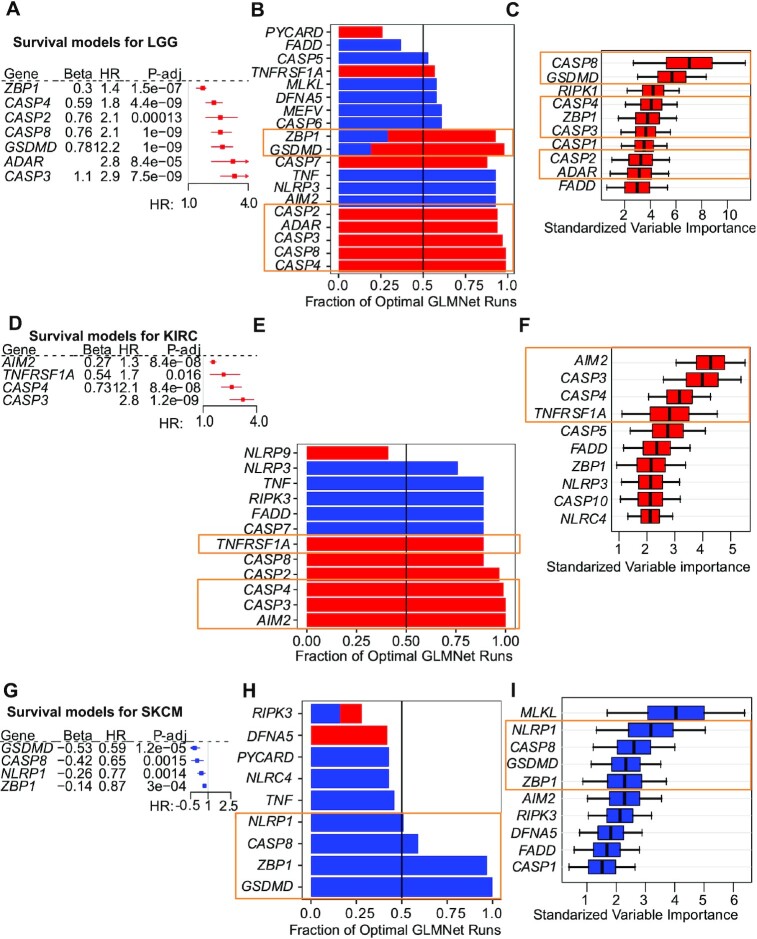
Multiple survival models identify key prognostic PANoptosis markers for LGG, KIRC and SKCM. (**A**) Forest plot for key PANoptosis genes whose high expression leads to a poor prognosis for LGG identified through univariate survival models. (**B**) PANoptosis genes with non-zero coefficients and the fraction of times they appeared during the 100 random runs of the GLMnet model for LGG. (**C**) Top 10 PANoptosis genes with highest prognostic relevance determined by the optimal RFS model for LGG. (**D**) Forest plot for key PANoptosis genes whose high expression leads to a poor prognosis for KIRC identified through univariate survival models. (**E**) PANoptosis genes with non-zero coefficients and the fraction of times they appeared during the 100 random runs of the GLMnet model for KIRC. (**F**) Top 10 PANoptosis genes with highest prognostic relevance determined by the optimal RFS model for KIRC. (**G**) Forest plot for key PANoptosis genes whose high expression leads to better prognosis for SKCM identified by univariate survival models. (**H**) PANoptosis genes with non-zero coefficients and the fraction of times they appeared during the 100 random runs of the GLMnet model for SKCM. (**I**) Top 10 PANoptosis genes with highest prognostic relevance determined by the optimal RFS model for SKCM. (A–I) Blue bars represent a negative coefficient (higher expression is beneficial for survival), and red bars represent a positive coefficient (higher expression is detrimental for survival). The orange boxes highlight the genes which are prognostic across the univariate, GLMNet and RFS survival models and were considered as the ‘Top’ PANoptosis markers. (B, C, E, F, H, I) The boxplots correspond to variable importance estimated using a subsampling approach.

We first examined the prognostic impact of the expression of individual PANoptosis genes for each tumor type. High expression of the majority of the PANoptosis genes in LGG corresponded to significantly worse survival prognosis (HR > 1) as observed from the univariate survival models (Figure 3A and Supplementary Figure S6A). We then used the elastic-net model as our generalized linear model (GLMnet) for survival analysis. However, since several of the PANoptosis genes had correlated expression profiles (Supplementary Figures S1A–C), the GLMnet model can result in a diverse set of genes with non-zero coefficients (either beneficial or detrimental to survival) for different initial random seeds (63). This was an important caveat that has not been accounted for by several previously published techniques using GLMnet models for cox regression analysis (5–13). To overcome this limitation, we ran our GLMnet model 100 times with 100 random seeds and 5-fold cross-validation to obtain the optimal model (in terms of cross-validation CI) during each run (Supplementary Figures S7A and B). Different PANoptosis genes appeared in the optimal model at different rates across the 100 runs (Figure 3B). We only considered those genes which appeared in at least 50% of the GLMnet models as prognostically relevant. Finally, we also built a non-linear random-forest survival analysis (RFS) model (64) with hyper-parameter optimization using a cross-validation technique (Supplementary Figure S7C). We then intersected the gene sets from each of these analyses to identify the key set of PANoptosis genes with prognostic impact for each of the cancer subtypes.

In the case of LGG, the GLMnet models suggested sensors and upstream regulators such as *ZBP1* and *ADAR* and effectors *CASP2*, *CASP3*, *CASP4*, *CASP8* and *GSDMD* had positive coefficients in the majority of the 100 runs (Figure 3B). However, the GLMnet models also indicated that genes such as *AIM2*, *NLRP3* and *TNF* had negative coefficients, suggesting that their expression would be beneficial for survival (Figure 3B) as per the GLMnet model. Since these genes either were not significant in the univariate model (Supplementary Figure S6A) or not in the top 10 genes identified by the RFS model (Figure 3C), they were not considered as key PANoptosis genes with prognostic impact for LGG. In contrast, *ZBP1*, *ADAR*, *CASP2*, *CASP3*, *CASP4*, *CASP8* and *GSDMD* were among the top 10 most important variables for the RFS model (Figure 3C) and were all present in the best GLMnet model (Supplementary Figure S8A) determined by cross-validation. We therefore identified these genes as the seven key PANoptosis markers, or ‘Top’ gene set, that are prognostically important for LGG.

The mean cross-validation (CV) performance of the GLMnet (CI = 0.801) and RFS (CI = 0.781) using both primary and secondary markers for LGG was slightly better than the GLMnet (CI = 0.786) and RFS (CI = 0.771) using only primary markers (Supplementary Table S3). Moreover, the survival models built using the ‘Top’ gene set achieved a mean CV performance of CI = 0.794 for GLMnet and CI = 0.774 for RFS, which is comparable to their respective primary plus secondary models (Supplementary Table S3 and S4). We further validated the prognostic value of these markers on an independent test set (GSE16011). The RFS model using both primary and secondary markers achieved a CI of 0.662, while the GLMnet model attained a CI of 0.649 (Supplementary Table S3). Similarly, using the ‘Top’ gene set the RFS model attained a CI of 0.672, while the GLMnet model achieved a CI of 0.615 (Supplementary Table S4). While there was a loss of performance with respect to the CV CI scores, the predictive capability of the models remained much higher than random. This loss in predictive capability could also be attributed to the relatively small size (20 samples) of the out-of-box validation set.

**Figure 4.**
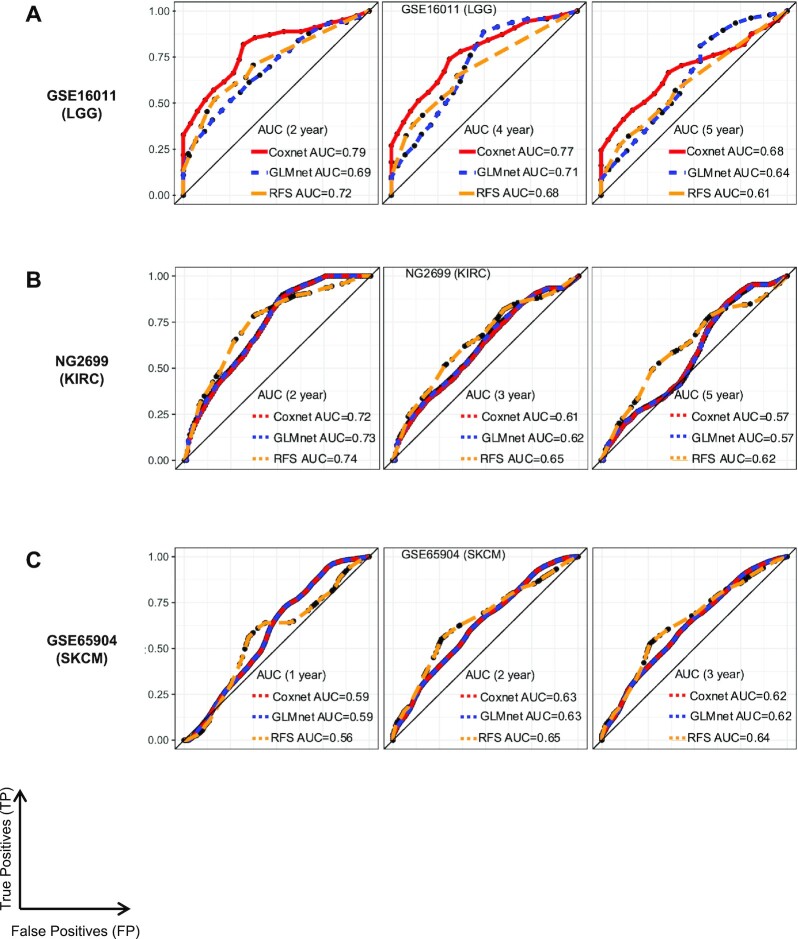
Survival models built using key PANoptosis markers predict survival on independent test sets. (**A**) Comparison of AUC metric at *t* ∈ {2,4,5} years between Coxnet, GLMnet and RFS survival models for LGG. (**B**) Comparison of AUC metric at *t* ∈ {2,3,5} years between Coxnet, GLMnet and RFS survival models for KIRC. (**C**) Comparison of AUC metric at *t* ∈ {1,2,3} years between Coxnet, GLMnet and RFS models for SKCM.

We also obtained AUC metrics at different time intervals (*t*) for the Coxnet, GLMnet and RFS models built using the ‘Top’ PANoptosis markers for LGG on the external validation set (GSE16011) (Figure 4A). The Coxnet model was the most accurate (AUC = 0.79 at *t* = 2 years, AUC = 0.77 at *t* = 4 years and AUC = 0.68 at *t* = 5 years) among the three different survival models (Figure 4A). Since a large majority of the events (death) in GSE16011 happened within the first 2 years, the predictive capability of the Coxnet model was maximal at that time point, i.e., it could accurately identify the at-risk patients. Over a longer time-period the predictive capability of the Coxnet model decreased but was higher than the GLMnet (AUC = 0.71 at *t* = 4 years and AUC = 0.64 at *t* = 5 years) and the non-linear RFS (AUC = 0.68 at *t* = 4 years and AUC = 0.61 at t = 5 years) models, respectively. This suggests that a linear combination of the seven PANoptosis markers (*ZBP1*, *ADAR*, *CASP2*, *CASP3*, *CASP4*, *CASP8* and *GSDMD*) was sufficient in accurately identifying the at-risk strata in LGG. Additionally, all the models achieved a predictive performance (AUC) better than random (AUC > 0.5) at all time points, showcasing the predictive performance of our proposed approach.

Similar analyses were performed for the other cancer types. For KIRC, the majority of PANoptosis genes corresponded to significantly worse OS when highly expressed (Figure 3D and Supplementary Figure S6B). From the GLMnet models (Figure 3E) and RFS model (Figure 3F), we identified sensors and upstream regulators such as *AIM2* and *TNFRSF1A* and effectors such as *CASP3* and *CASP4* as the key PANoptosis genes associated with worse survival prognosis across multiple survival analysis models for KIRC. All the aforementioned genes were part of the best GLMnet, which had the highest CV CI among 100 runs (Supplementary Figure S8B). The GLMnet and RFS models for KIRC using both primary and secondary markers had similar predictive capability in the CV dataset as the models that were built using only primary markers (Supplementary Table S3) or the above-mentioned ‘Top’ four key PANoptosis genes (Supplementary Table S4). Furthermore, on the independent validation test set, the different survival models (both GLMnet and RFS) had similar performances as the CV CI (≅ 0.65) with a slight loss in performance of the survival models built using only the ‘Top’ four PANoptosis markers (CI ≅ 0.62), demonstrating the efficient prognostic and generalization capability of the survival models for KIRC. For KIRC, the non-linear RFS model performed the best with respect to the AUC metric (AUC = 0.74 at *t* = 2 years, AUC = 0.65 at *t* = 3 years and AUC = 0.62 at *t* = 5 years) among the Coxnet, GLMnet and RFS models (Figure 4B). In the NG2699 dataset, the patients at risk generally die within the first five years, while a large proportion of the patients remain alive beyond this time and hence were censored in the analysis. The predictive performance (AUC) of all the survival models decreased over time (Figure 4B), though it was always better than random (AUC > 0.5).

For SKCM, from the univariate models, we observed that the expression of the majority of the PANoptosis genes was beneficial for survival (HR < 1 and adjusted *P*-value < 0.05) (Figure 3G and Supplementary Figure S6C), and specific genes were identified that were consistent with the other survival models with regards to their prognostic significance (Figure 3G). For SKCM, these included *ZBP1, NLRP1, CASP8* and *GSDMD* (Figure 3H), all of which were beneficial for survival when included in the best GLMnet model (Supplementary Figure S8C). The top 10 genes identified in the RFS model for SKCM included *ZBP1*, *NLRP1*, *CASP8* and *GSDMD* (Figure 3I), which we selected as the ‘Top’ gene set based on their importance in each of the survival models. The mean CV performance of GLMnet (CI = 0.670) and RFS (CI = 0.626) using both primary and secondary markers (Supplementary Table S3) were similar to the GLMnet (CI = 0.673) and RFS (CI = 0.612) using only primary markers. Additionally, the performance of GLMnet (CI = 0.664) and RFS (CI = 0.615) using the ‘Top’ 4 key PANoptosis genes was similar to those of models built using primary and secondary markers (Supplementary Table S4). Moreover, we validated the general applicability of our different survival models on two independent test sets for SKCM. These test sets were obtained through GSE65904 and GSE22155. Each of the Coxnet, GLMnet and RFS models achieved a CI score of ≅0.6 on the two out-of-box datasets, comparable to their CV performance (Supplementary Table S3 and Supplementary Table S4). Furthermore, the efficiency of the three different survival models built using the ‘Top’ 4 PANoptosis markers (*ZBP1, NLRP1, CASP8* and *GSDMD*) was consistent, AUC ≅ 0.6, on the independent validation set (GSE65904) at time *t* ∈ {1, 2, 3} years (Figure 4C). As an additional confirmation, we found that PANoptosis score was significantly prognostic in each of the test sets when we built multivariate Coxnet survival models including patient clinical features such as age, gender, grade or stage of cancer in combination with the PANoptosis score for LGG, KIRC and SKCM cancer subtypes (Supplementary Figure S3).

Overall, using a consensus of diverse survival analysis models with adequate predictive capabilities, we found a refined set of potentially targetable PANoptosis genes with clinical relevance for OS. Further, we built and validated efficient prognostic survival models for each of the three cancer types of interest.

### Single cell level evidence for PANoptosis in LGG and SKCM scRNA-seq datasets

**Figure 5.**
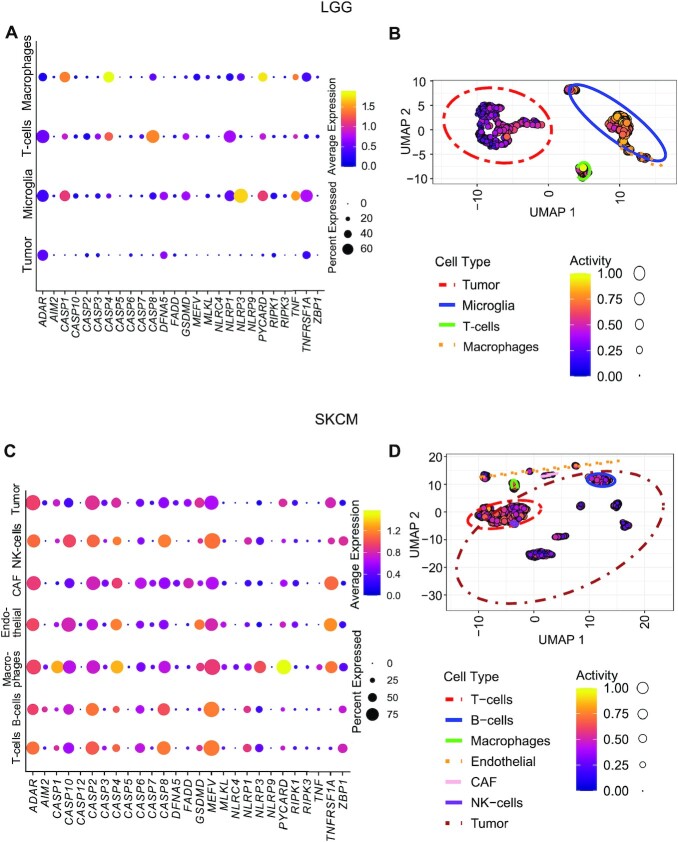
Single cell transcriptomics provides evidence for PANoptosis in individual cells in LGG and SKCM datasets. (**A**) Expression profiles of PANoptosis genes across different cell types in the LGG dataset. (**B**) PANoptosis activity across different cell types in the LGG dataset estimated using ssGSEA. (**C**) Expression profiles of PANoptosis genes across different cell types for the SKCM dataset. (**D**) PANoptosis activity across different cell types in the SKCM dataset estimated using ssGSEA.

The TCGA data analyzed to form our training set and the data for our out-of-box test sets included bulk level RNAseq data for the tumor types of interest. We next sought to understand the relevance of PANoptosis gene expression at the single cell level by analyzing available scRNA-seq datasets. In an LGG single cell transcriptomics dataset (47), we observed that the majority of PANoptosis markers had low average and percentage expression in tumor cells (Figure 5A). However, in immune cell types, specifically T-cells and microglia, several PANoptosis markers were either highly expressed or expressed in a high percentage of the corresponding cells (Figure 5A). These results were further reflected in the PANoptosis activities estimated for every cell using the ssGSEA technique and visualized through UMAP; we observed that there was less PANoptosis activity in the tumor cells compared to immune cells (Figure 5B).

In contrast, in the SKCM dataset (48), several of the PANoptosis markers were expressed in a high percentage of tumor cells (Figure 5C). However, the average expression of most PANoptosis markers was lower in tumor cells when compared to their expression across immune (T-cells, B-cells, NK-cells and macrophages) or stromal (endothelial) cells (Figure 5C). We again observed less PANoptosis activity in the tumor cells when compared to the immune and stromal cells in the SKCM dataset (Figure 5D), highlighting this consistent observation at single cell resolution. Furthermore, the tumor cells in the SKCM dataset were obtained from six different patients and had significantly different transcriptomic profiles, leading to disjointed clusters (Figure 5D).

### Proof-of-concept: PANoptosis causes cell death in melanoma cancer cell lines

Among the three different cancer types where PANoptosis had significant prognostic impact, higher expression of key cell death molecules involved in PANoptosis showed significant positive association with survival probability in patients with melanoma (SKCM). This suggests that inducing the expression of the key cell death molecules associated with these cancers may lead to PANoptosis and be a beneficial treatment strategy for these cancers.

ZBP1 is a master regulator of PANoptosis. In murine models, sensing of nucleic acids by the Zα domain of ZBP1 leads to its receptor-interacting protein homotypic interaction motif (RHIM) domain interacting with the corresponding RHIM domain of RIPK3 to drive cell death (75–78). ZBP1 has previously been identified as the key upstream sensor capable of activating the NLRP3 inflammasome, caspase-8, caspase-7, GSDMD and MLKL to promote PANoptosis (14,17,77), and we identified here that its expression was positively associated with increased survival in SKCM. Thus, we evaluated whether treatments that target the ZBP1-mediated PANoptosis pathway would be effective to induce cell death in melanoma cancer cell lines.

**Figure 6.**
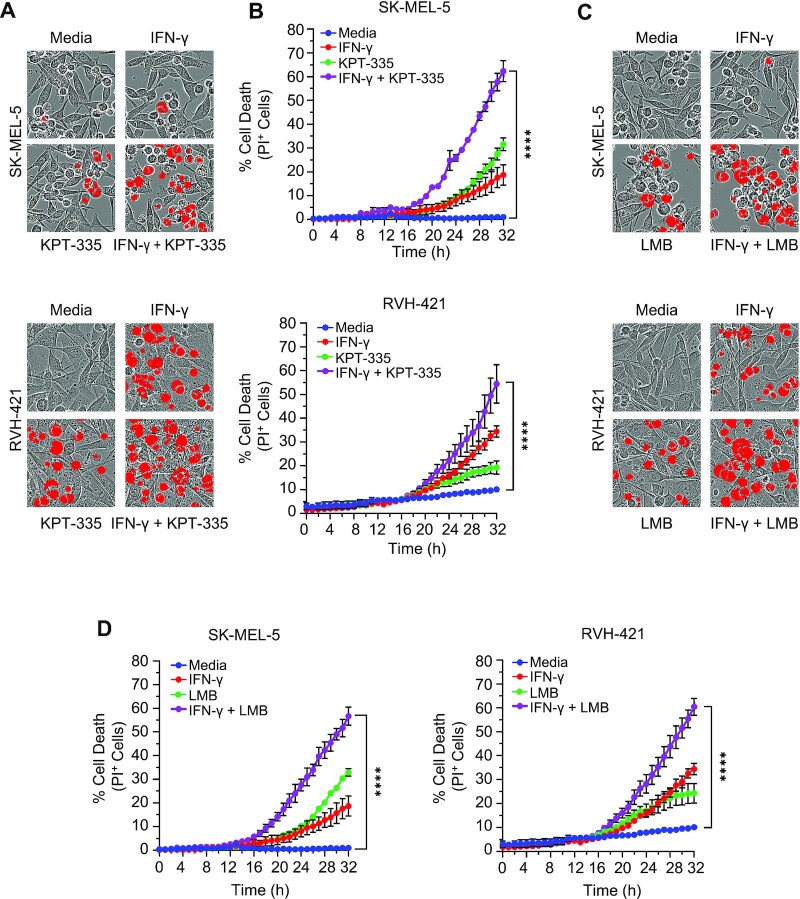
Proof-of-concept – activation of ZBP1-mediated PANoptosis induced cell death in melanoma cells. (**A**) Representative images of cell death by propidium iodide (PI) staining in SK-MEL-5 (top) and RVH-421 (bottom) cell lines treated with KPT-335 in the presence or absence of IFN-γ. Red mask denotes dead cells. (**B**) Quantification of cell death by PI staining in SK-MEL-5 (top) and RVH-421 (bottom) cell lines treated with KPT-335 in the presence or absence of IFN-γ. (**C**) Representative images of cell death by PI staining in SK-MEL-5 (top) and RVH-421 (bottom) cell lines treated with leptomycin B (LMB) in the presence or absence of IFN-γ. (**D**) Quantification of cell death by PI staining in SK-MEL-5 (left) and RVH-421 (right) cell lines treated with LMB in the presence or absence of IFN-γ. ****P-value < 0.0001.

We selected two human melanoma cell lines, SK-MEL-5 and RVH-421, based on their diversity in terms of gender, tumor type as well as sample collection site. Moreover, these two cell lines had a high PANoptosis score (Supplementary Figure S8D) estimated using the ssGSEA technique, thereby suggesting a higher possibility of cell death through the induction of PANoptosis. Previously, it had been shown that nuclear export inhibitors induce ZBP1-dependent PANoptosis in myeloid cells in the presence of interferons (IFNs) (17). Therefore, to study the ZBP1-dependent cell death in cancer cells, we treated the two different melanoma cells lines with nuclear export inhibitors KPT-335 or leptomycin B (LMB) with or without IFN-γ and examined cell death. Cell death was substantially increased in melanoma cell lines treated with KPT-335 plus IFN-γ or LMB plus IFN-γ compared with individual triggers or media alone (Figure 6A–D). We performed additional molecular characterization in the RVH-421 cell line and found that these combination treatments induced expression of *ZBP1* and led to the activation of PANoptotic markers, including caspase-1, GSDMD, GSDME, caspase-8, caspase-3 and caspase-7 (Supplementary Figure S9). Together, these results indicate that treatments which promote the upregulation of *ZBP1* can increase cell death in melanoma cell lines. This suggests that the key PANoptosis molecules identified by our framework are potential therapeutic targets in cancer and can drive the next generation of clinical treatments.

## DISCUSSION

PCD is important in the elimination of infected or damaged host cells, and its dysfunction plays an important role in cancers (79). While apoptosis is often considered to be key in tumor suppression (80), the relationship between cancer and inflammatory PCD pathways such as pyroptosis, necroptosis and PANoptosis is complex. Though the induction of inflammatory cell death can inhibit the occurrence and development of tumors, pyroptosis and necroptosis can also propagate tumorigenesis (81). While previous studies have evaluated the roles of pyroptosis, apoptosis and necroptosis independently in cancers (5–8), increasing evidence suggests there is also a critical role for PANoptosis in cancers (17,18,28). Thus, understanding the role of PANoptosis in cancer is of paramount importance for developing more efficient patient stratification systems to guide tumor-specific therapeutic strategies (82).

Here, we have established a systematic framework to elucidate the prognostic impact of PANoptosis gene expression based on a pancancer cohort of 32 distinct cancer types from the TCGA. We established a PANoptosis gene signature-based patient stratification system that informs differential associations with OS. We further found three cancer subtypes where PANoptosis has a significant prognostic impact and characterized the specific PANoptosis genes of clinical relevance through a two-step filtering procedure encompassing (a) differential expression analysis and (b) survival analysis. These analyses allowed us to perform a comprehensive validation of the prognostic relevance of PANoptosis in a series of out-of-box unseen test sets of relevant cancer lineages. We further applied our identified biomarkers in a proof-of-concept experiment to illustrate that PANoptosis caused cell death in melanoma cells.

We initially considered 27 genes in our PANoptosis gene set based on evidence from the literature that these genes play a role in inducing PANoptosis in response to various pathogens and stimuli (16,17,31–33,71,83,84). As more evidence of crosstalk and functional redundancies emerge among the molecular components of pyroptosis, apoptosis and necroptosis (85,86), and more molecules of relevance are identified (87), the PANoptosis gene set may need to be expanded further to fully understand its potential for prognostic prediction. Using our computational framework, we identified cancer types where increased expression of the PANoptosis genes is significantly beneficial for OS (SKCM) and where increased expression of the PANoptosis genes is significantly detrimental for OS (LGG and KIRC). Recent work using a relatively simplistic modeling technique (LASSO) shows that individually, pyroptosis and necroptosis are prognostic for SKCM, stomach adenocarcinoma (STAD), LGG and KIRC cancers based on the TCGA cohort (56,88–90). Moreover, immunologically cold tumors for LGG and KIRC have better survival prognosis in comparison to immunologically hot tumors (52,53); this has generally been attributed to tumor signaling including pathways known to be associated with immune suppression such as transforming growth factor beta (*TGFB*) (91). These results align with our finding that high expression of genes associated with inflammatory cell death, PANoptosis, is detrimental in these tumors.

While previous modeling approaches have attempted to understand the impact of individual inflammatory PCD pathways on cancer prognosis (56,88–90), our modeling approach overcomes key limitations faced in previous studies. The majority of these approaches filter the initial gene set using a LASSO-based regularized cox regression model to determine a reduced set of genes as the final prognostic set (56,88–90). However, there is a strong correlation between the genes in the initial gene set, and the LASSO-based linear survival models can suffer from degenerate solutions, i.e., the model will identify a different set of genes as important and with non-zero coefficients when built with different random initializations (92). To circumvent this limitation, we used an elastic-net regularized cox regression analysis model (GLMnet), which can attenuate some of the drawbacks of the LASSO model through an L_2_ regularization term, and we ran our GLMnet models 100 times. Then, the relevant prognostic markers were identified as those which have non-zero coefficients in at least 50% of the models. Furthermore, by using a non-linear RFS model, our approach can handle correlated sets of genes. This is because RFS employs a random sampling of the correlated genes to build its decision trees and ensembles them. The RFS model also provides variable importance with confidence intervals, i.e., it can rank the genes based on their importance for survival.

In this work, we aimed to determine the role of PANoptosis in cancer patient stratification and establish its clinical relevance, as well as devise a systematic mechanism to identify key therapeutic targets for cancers of interest. However, in-depth molecular characterization of these PANoptosis clusters is needed. Additionally, characterization of the mutational profile, genomic stability, immune composition and pathway activity of the patients stratified into groups should be performed. This has previously been useful when using a gene set-based signature to understand the differential prognosis with respect to the gene set of interest (8,52), and it can also provide complimentary evidence driving the prognosis. For instance, the role of immune composition (percentage of different immune cell types identified through deconvolution of bulk RNA-Seq data) has been found to complement the prognostic value of the inflammasome clusters (8). Similarly, the enrichment of tumor intrinsic pathways may determine the prognostic and predictive value of the immunologic constant of rejection signature when used for stratification of patient tumors (8,52). Furthermore, differential analysis through network-based techniques such as differential network analysis (93–95) and master regulator analysis (96–98) can provide insights about the molecular mechanisms underlying PANoptosis activity in modulating innate immunosurveillance in the cancer lineages of interest.

Additionally, further studies using proteomic data will also be important. A limitation of our current work is that the signature is based on gene expression profiles of PANoptosis genes. A high gene expression does not necessarily denote a high activation of its corresponding protein. Post-transcriptional and post-translational modifications can impact the protein expression and activity of PANoptosis genes. However, recent estimates of correlations between whole genome mRNA levels and protein abundance by state-of-the-art proteome quantification techniques are >0.8 (99), suggesting gene expression can be informative for protein expression. Finally, through analysis of two scRNA-seq datasets, we illustrated PANoptosis activity at single cell resolution. In both the datasets, we consistently observed reduced expression of PANoptosis genes in the tumor cells within the tumor microenvironment, and further studies will be needed to determine whether this is suggestive of intrinsic resistance to PCD. In the future, spatial transcriptional analysis can also be employed which could enable characterization of human tumors undergoing PANoptosis with higher fidelity, and microscopy can be used to confirm the activation of PANoptosis in individual cancer cells. Recent studies have successfully observed PANoptosome formation in single cells using confocal microscopy (16,84,100), and specifically in response to the therapeutic combination of a nuclear export inhibitor and IFN (100), which we used in this study to activate ZBP1-mediated PANoptosis. The activated PANoptosis biochemical markers observed in the RVH-421 cell line indicate that a multiprotein PANoptosome is forming, and imaging these cells in the future would provide important information about the state of PANoptosis in human cancer cells.

Overall, our work provides a systematic framework to analyze and identify key innate immune biomarkers that could be targeted to improve patient outcomes in cancer. By analyzing the prognostic value of PANoptosis clusters and determining the minimal gene set responsible for the majority of the prognostic capability, our models provide a solid foundation for the identification of targetable molecules that can be modulated in cancer therapies.

## DATA AVAILABILITY

All code and relevant data are publicly available on Mendeley (doi: 10.17632/5drb9c5y9h.2).

## Supplementary Material

zcac033_Supplemental_File
