# Supplementary material for: Pancancer transcriptomic profiling identifies key PANoptosis markers as therapeutic targets for oncology
Source: NAR Cancer. 2022 Nov 1;4(4):zcac033. doi: 10.1093/narcan/zcac033 (PMC9623737; doi:10.1093/narcan/zcac033)
Supplement: zcac033_Supplemental_File [file zcac033_supplemental_file.pdf]

Supplemental Data

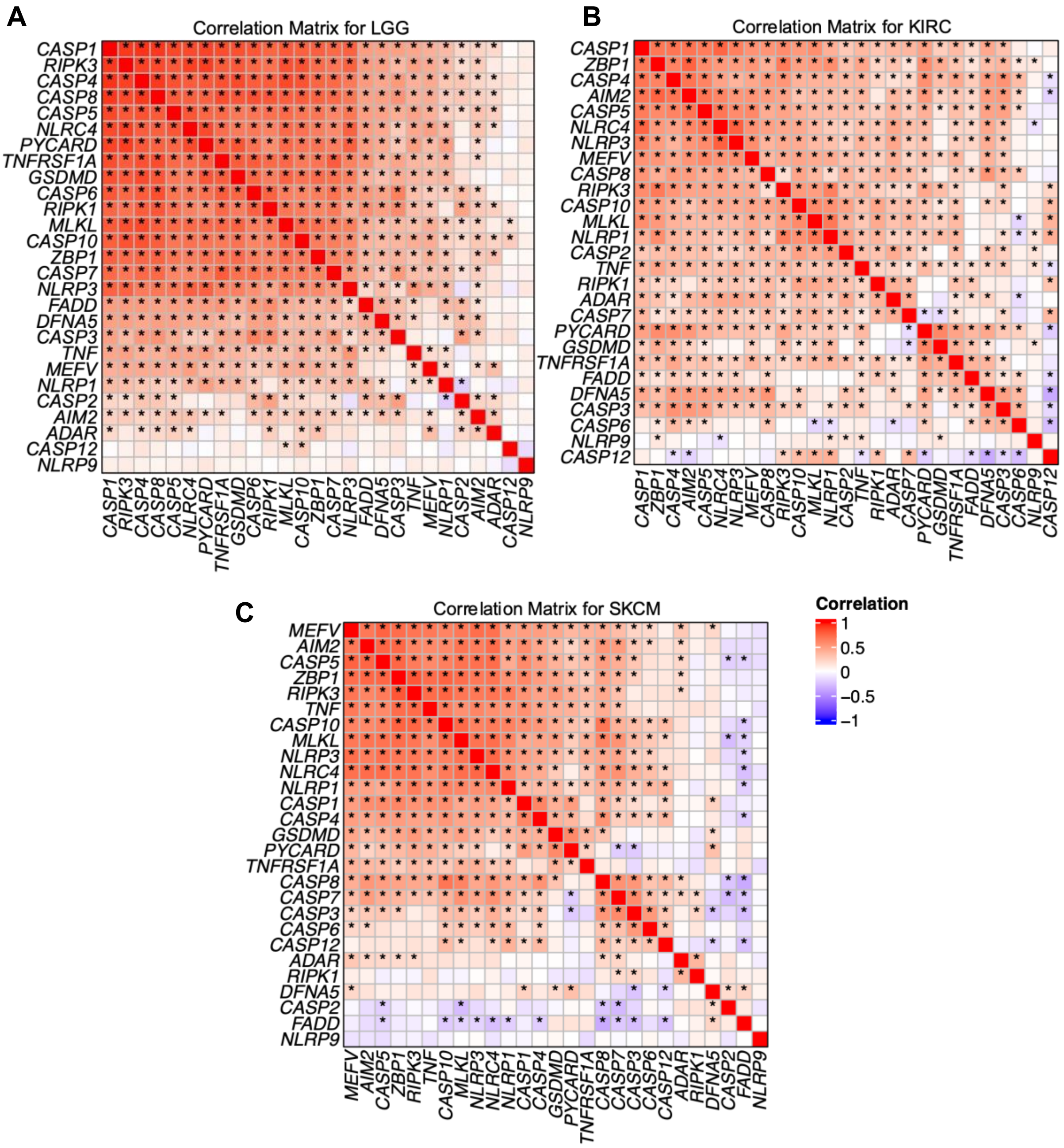

Supplementary Figure 1: **Expression of PANoptosis genes is correlated**

A-C) Correlation matrices of the PANoptosis genes using the expression profiles of these genes in LGG (A), KIRC (B) and SKCM (C) cancers. Correlations are estimated using Pearson correlation. * represents a statistically significant correlation.

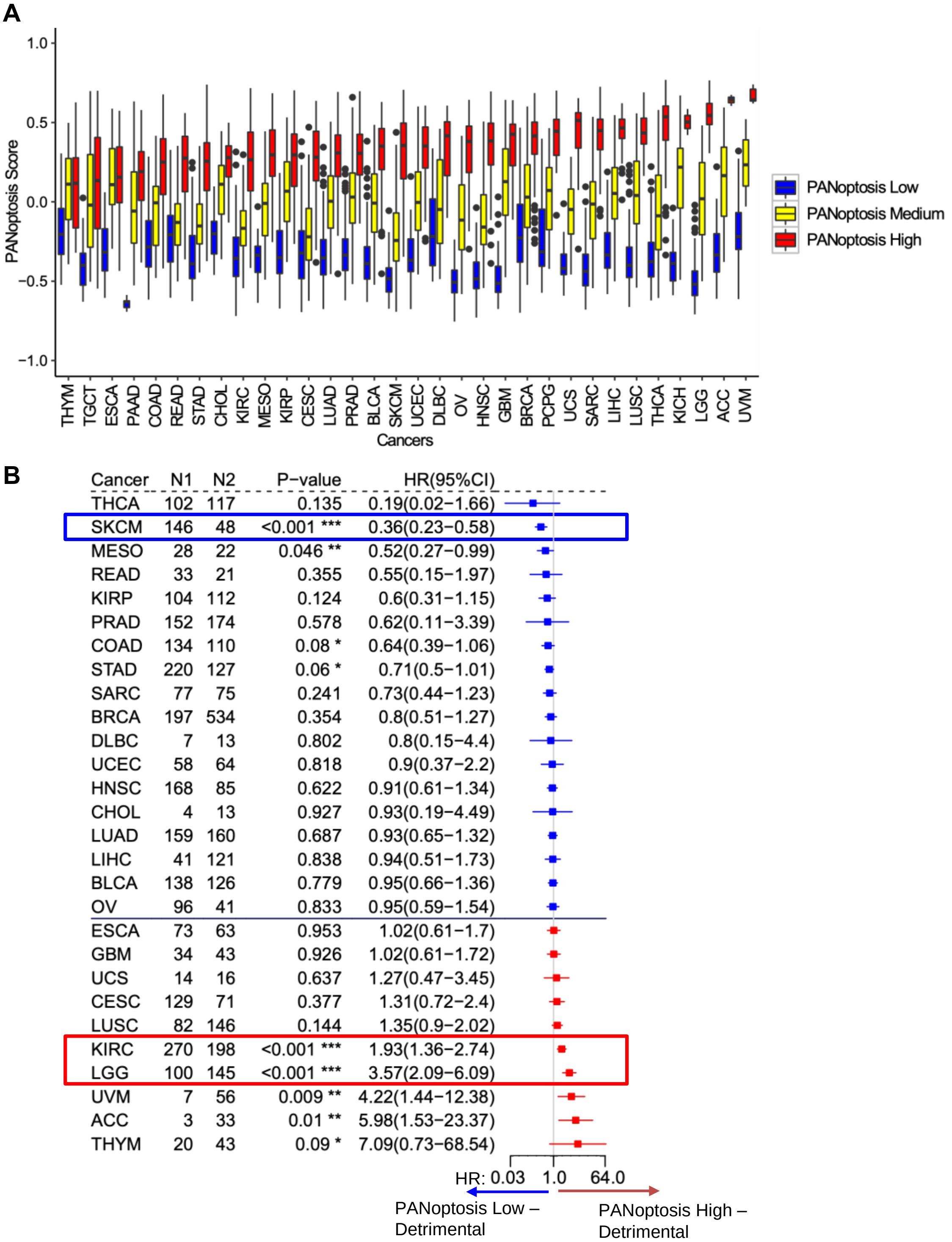

Supplementary Figure 2: **PANoptosis clustering in pancancer prognosis**

A) Boxplots of PANoptosis scores bifurcated across the three PANoptosis clusters for each of the 32 different cancer types in TCGA. **B)** Forest plot showing N1 = number of samples in PANoptosis high cluster, N2 = number of samples in PANoptosis low cluster, *P*-value and hazard ratio (HR) with 95% CI for overall survival (OS) when comparing PANoptosis high versus PANoptosis low clusters for each cancer type. Cancer types PCPG, PAAD, TGCT and KICH (**Table 1**) are excluded from the plot because the confidence interval of HR ranged from 0 to infinite due to the low number of death events in these cancer types. The blue box highlights SKCM, where PANoptosis high has significant beneficial association with OS. The red box highlights LGG and KIRC, where PANoptosis high has a significantly detrimental association with OS.

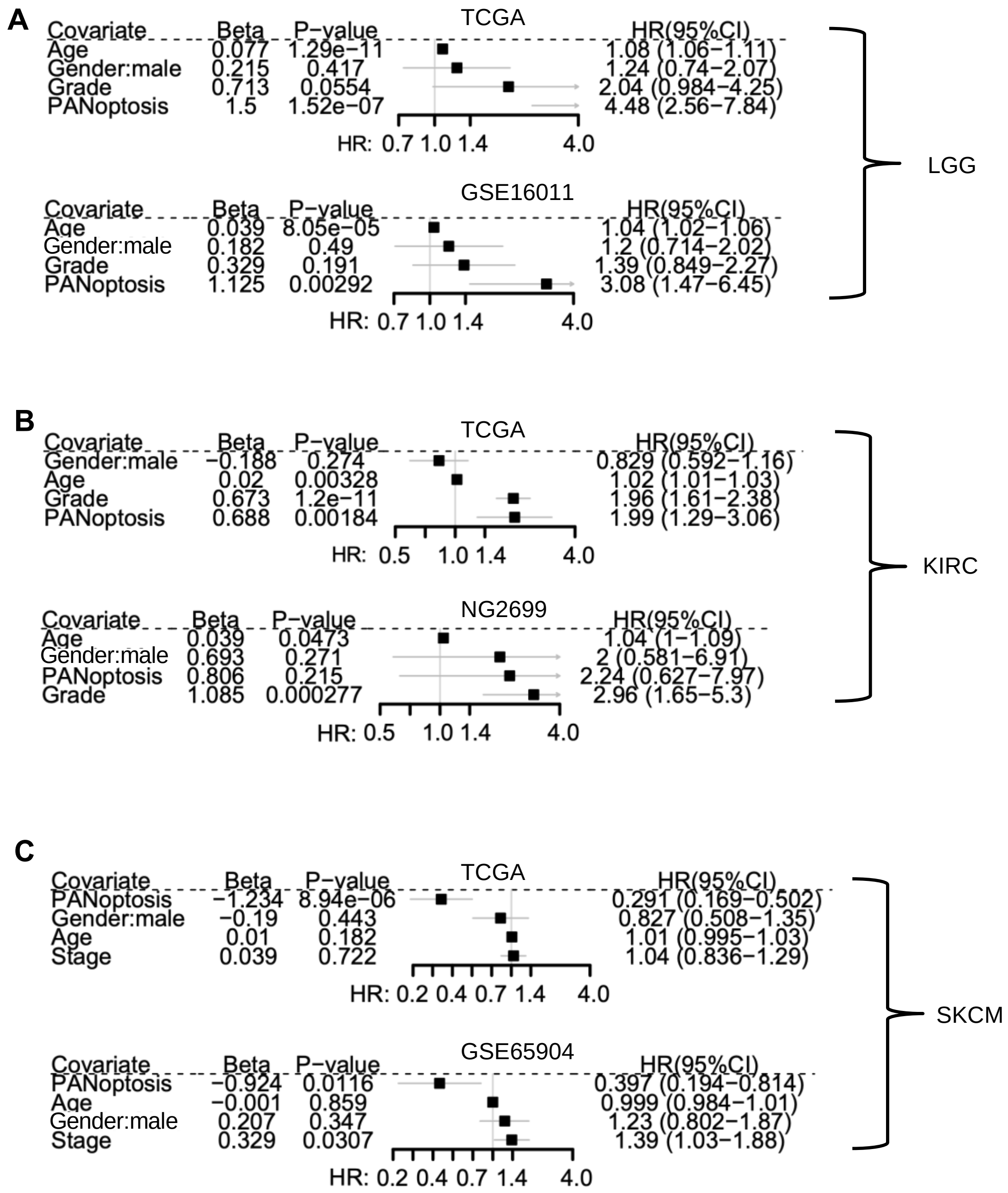

Supplementary Figure 3: **PANoptosis score is an independent factor for survival prediction**

A) Forest plot showing PANoptosis score was significantly prognostic in TCGA (top) as well as the independent test set GSE16011 (bottom) through multivariate Coxnet models for LGG cancer type. **B)** Forest plot showing PANoptosis score was significantly prognostic in TCGA (top) and followed a similar trend for independent test set NG2699 (bottom) through multivariate Coxnet models for KIRC cancer subtype. **C)** Forest plot showing PANoptosis score was significantly prognostic in TCGA (top) and the test set GSE65904 (bottom) through multivariate Coxnet models for SKCM cancer type.

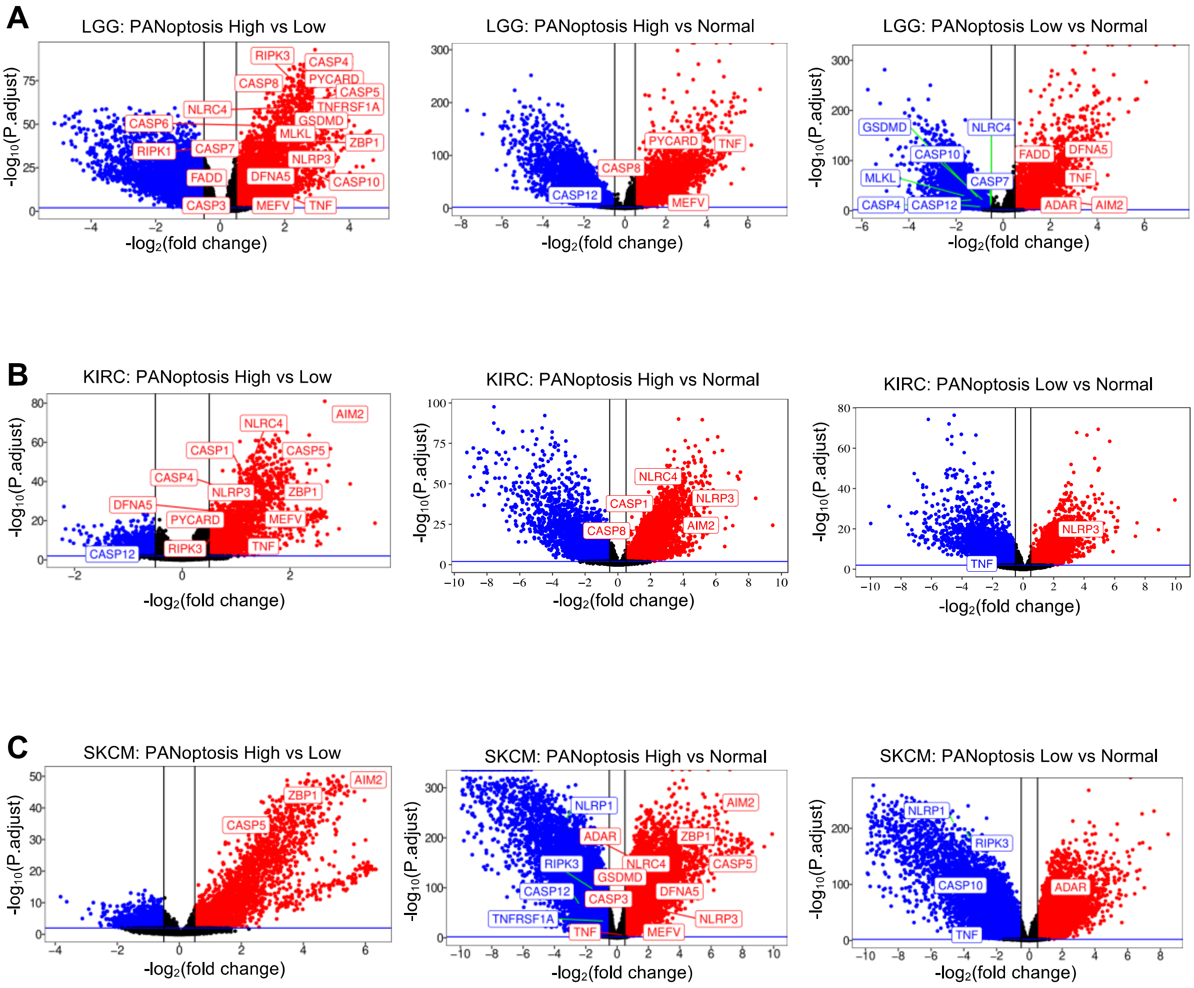

Supplementary Figure 4: Differential expression patterns of PANoptosis markers when comparing PANoptosis high, low and normal samples

A-C) Volcano plots highlighting differentially expressed genes for PANoptosis high vs PANoptosis low (left), PANoptosis high vs normal (middle) and PANoptosis low vs normal (right) samples in LGG (A), KIRC (B) and SKCM (C) cancer types. The differentially expressed PANoptosis markers are highlighted in the volcano plot. Genes in red are overexpressed while genes in blue are downregulated in the comparison condition. Here, 'Normal' samples correspond to those samples obtained from GTEx.

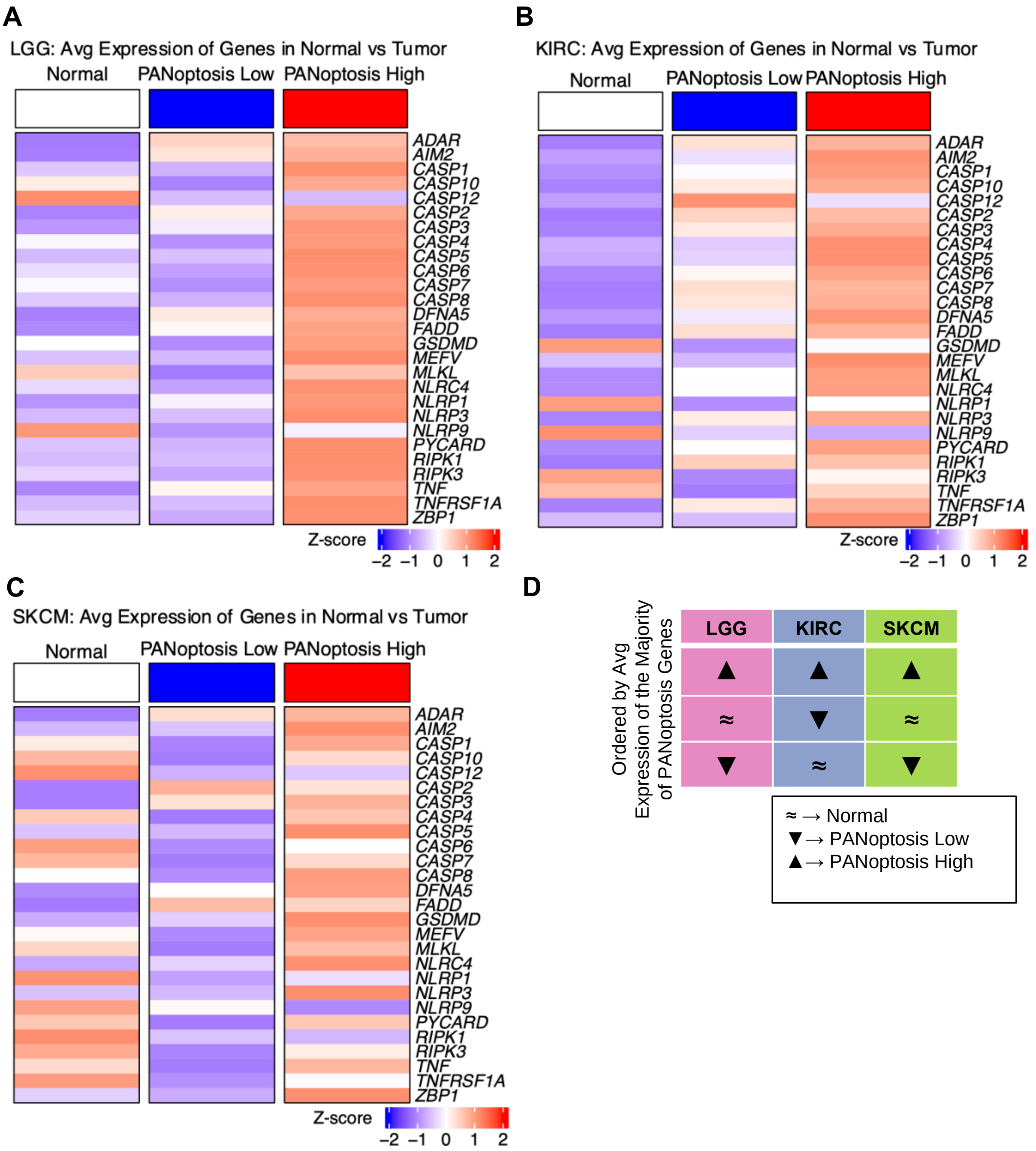

Supplementary Figure 5: Average expression pattern of PANoptosis markers
A-C) Average expression of PANoptosis markers in normal, PANoptosis low and PANoptosis high samples scaled to Z-scores for LGG (A), KIRC (B) and SKCM (C). **D)** A visual representation highlighting how the expression in PANoptosis high, PANoptosis low and normal categories can be ordered based on expression of the PANoptosis markers.

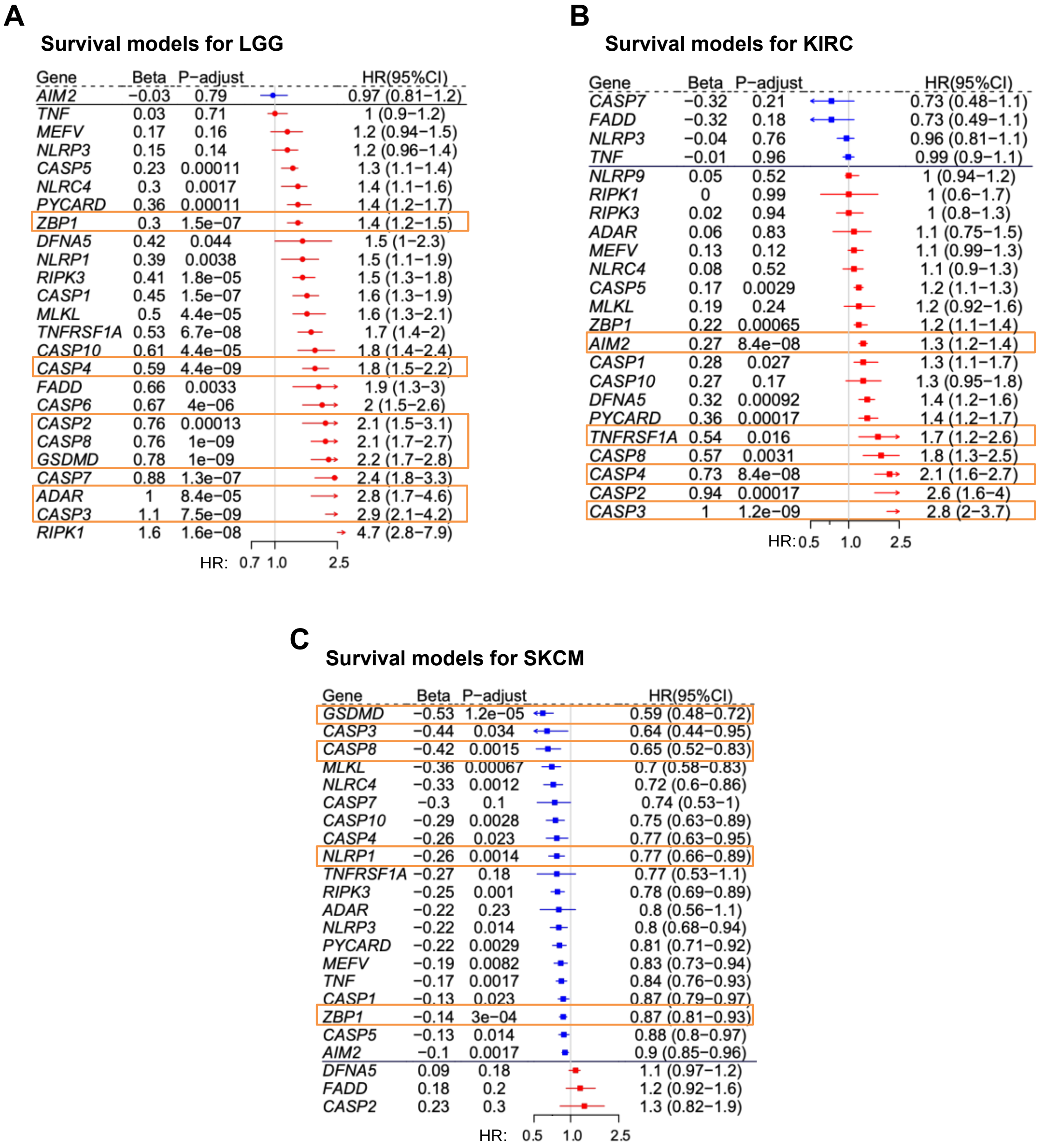

Supplementary Figure 6: Univariate survival models for LGG, KIRC and SKCM

A-C) Univariate survival models based on primary plus secondary PANoptosis genes for LGG (A), KIRC (B) and SKCM (C). Blue bars represent a negative coefficient (higher expression is beneficial for survival), and red bars represent a positive coefficient (higher expression is detrimental for survival). The orange boxes highlight the genes which are prognostic across the univariate, GLMNet and RFS survival models and were considered as the 'Top' PANoptosis markers.

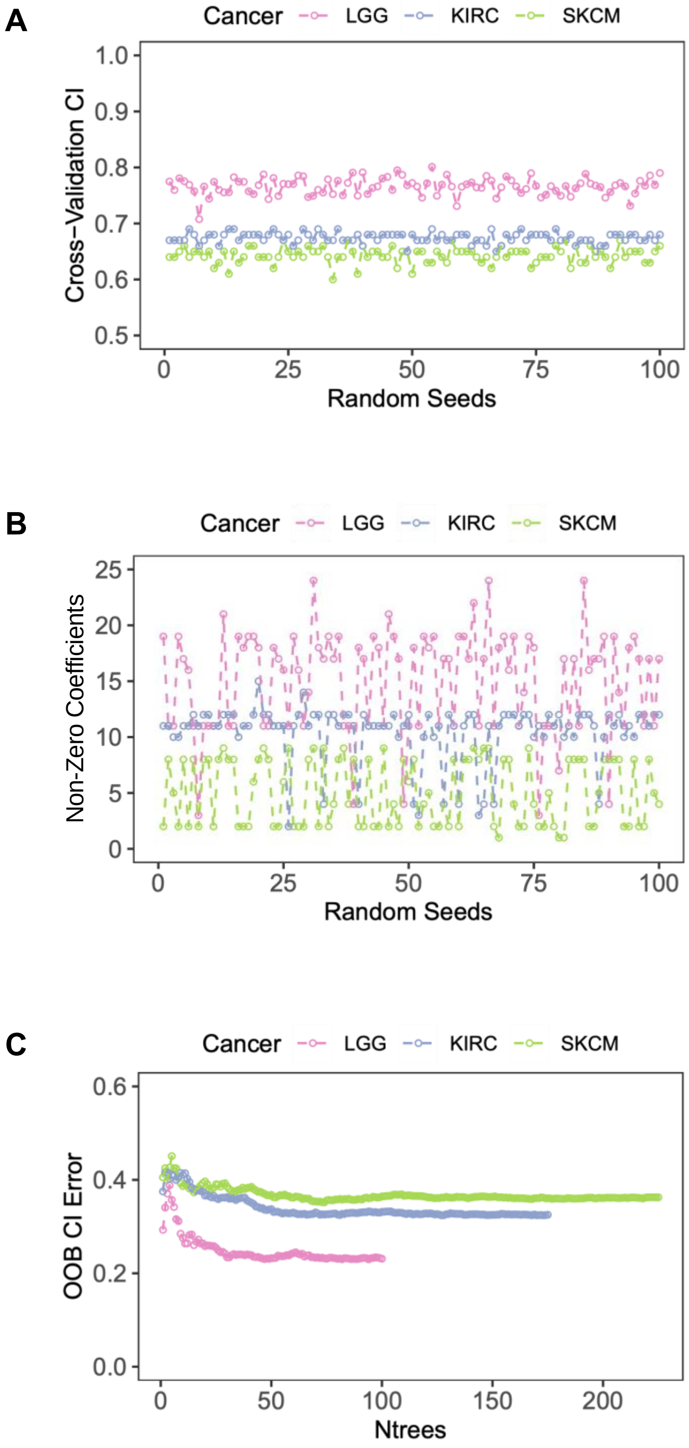

Supplementary Figure 7: Tuning hyperparameters of different survival models

A) Cross-validation CI for 100 runs of GLMnet for each of the three cancers of interest. **B)** Number of non-zero coefficients for the 100 runs of GLMnet for each of the three cancers. **C)** Performance of RFS model as a function of hyperparameter number of trees.

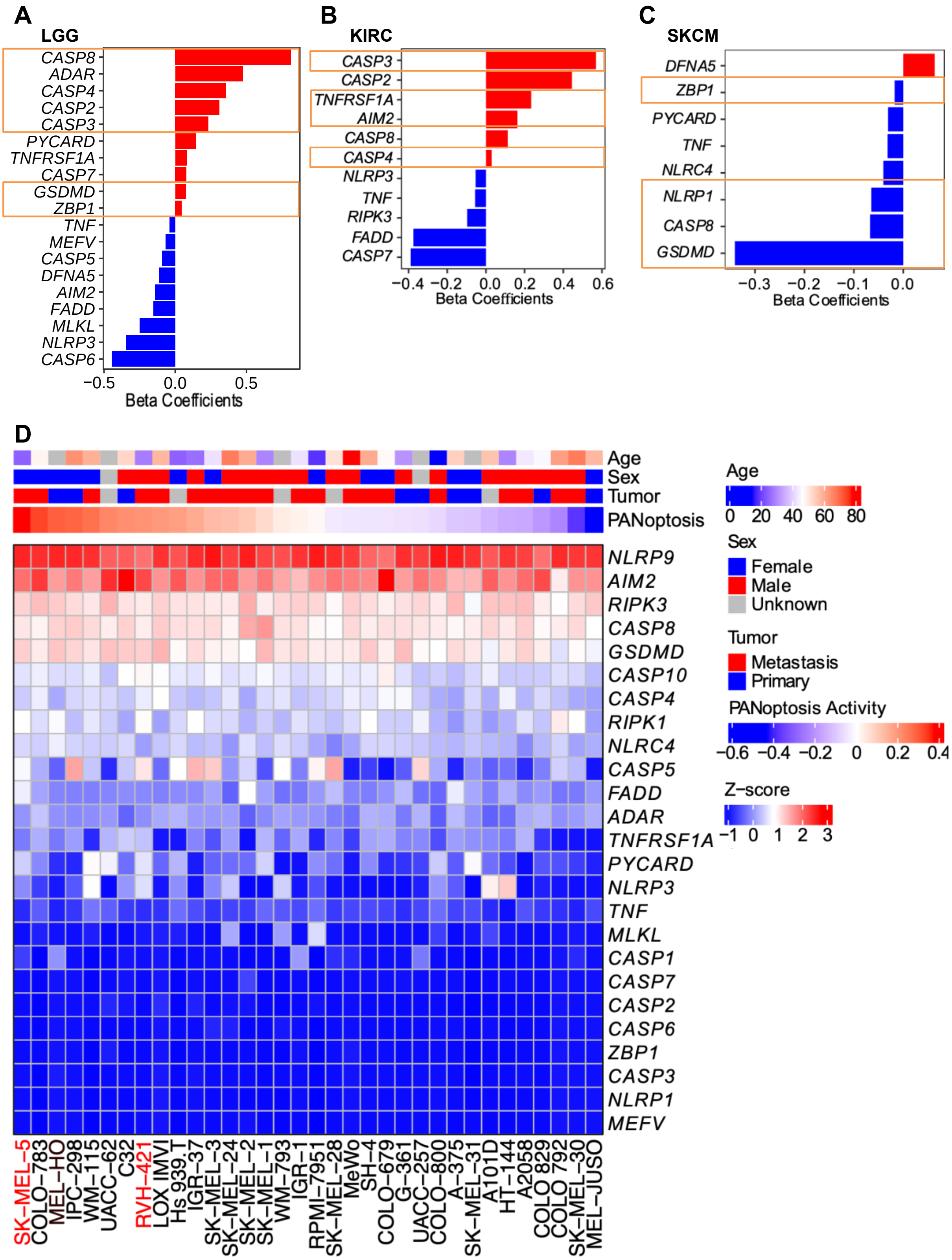

Supplementary Figure 8: Multiple survival models identify key prognostic PANoptosis markers and PANoptosis gene expression across SKCM cell lines

A-C) Optimal GLMnet models for LGG (A), KIRC (B) and SKCM (C). **D)** Scaled expression of the PANoptosis genes across the 34 melanoma cancer cell lines. Clinical characteristics of the cancer cell lines such as age, sex and tumor type are also highlighted in the heatmap.

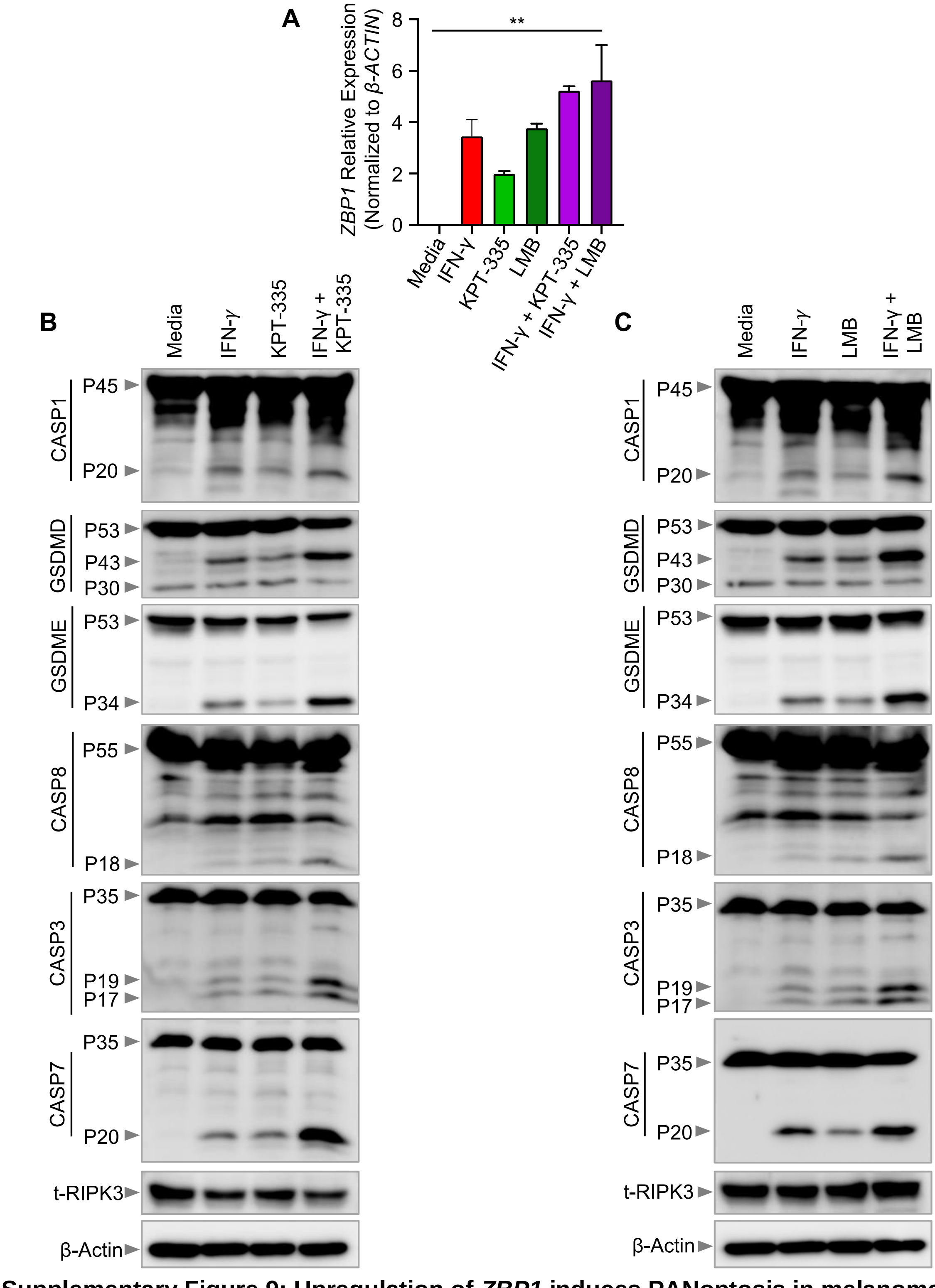

Supplementary Figure 9: Upregulation of *ZBP1* induces PANoptosis in melanoma cells
A) Real-time PCR analysis of *ZBP1* expression in RVH-421 cells treated with IFN-γ, KPT-335, leptomycin B (LMB) or combination treatment for 32 h. **B,C)** Western blot analysis of PANoptosis components in RVH-421 cells treated with IFN-γ, KPT-335, LMB or combination treatment for 32 h. Blots for pro- (P45) and activated (P20) caspase-1 (CASP1), pro- (P53) and activated (P30) gasdermin D (GSDMD), pro- (P53) and activated (P34) gasdermin E (GSDME), pro- (P55) and cleaved caspase-8 (CASP8; P18), pro- (P35) and cleaved caspase-3 (CASP3; P19 and P17), pro- (P35) and cleaved caspase-7 (CASP7; P20), and total RIPK3 (t-RIPK3) are shown, with β-actin included as the loading control. ***P*-value < 0.01.

Geneset	Sensors and Upstream Regulators	Adaptors	Effectors
PANoptosis	*ADAR, AIM2, MEFV, NLRC4, NLRP1, NLRP3, NLRP9, TNFRSF1A, ZBP1*	*FADD, PYCARD*	*CASP1, CASP10, CASP12, CASP2, CASP3, CASP4, CASP5, CASP6, CASP7, CASP8, DFNA5, GSDMD, MLKL, RIPK1, RIPK3, TNF*
Pyroptosis	*ADAR, AIM2, MEFV, NAIP, NLRC4, NLRP1, NLRP3, NLRP6, NLRP9, ZBP1*	*PYCARD*	*CASP1, CASP4, CASP5, CASP8, DFNA5, GSDMA, GSDMB, GSDMC, GSDMD*
Necroptosis	*ZBP1*		*CASP8, MLKL, RIPK3*
Apoptosis	*APAF1, BAX, BAK, DCN, DIABLO,FAS,TNFRSF1A*	*FADD*	*CASP2, CASP3, CASP6, CASP7, CASP8, CASP9, CASP10, CASP12, RIPK1, TNF*

Supplementary Table 1: Key programmed cell death genes considered in analyses.

	PANoptosis High (↑)	PANoptosis Low (↑)	Normal (↑)
LGG	*NLRC4, ZBP1, TNF, CASP1, AIM2, CASP5, MEFV, NLRP3, CASP7, DFNA5, FADD, PYCARD, CASP3, CASP4, CASP8, NLRP1, RIPK3, CASP2, CASP6, GSDMD, RIPK1, TNFRSF1A*	*ADAR, AIM2*	*CASP10, CASP12, MLKL*
KIRC	*NLRC4, ZBP1, CASP1, AIM2, CASP5, MEFV, CASP10, CASP7, FADD, DFNA5, NLRP3, CASP3, CASP4, ADAR, PYCARD, CASP8, CASP2, RIPK1, MLKL, TNFRSF1A*	*CASP12, CASP7, FADD, RIPK1, CASP2, CASP8, ADAR, CASP3*	*TNF, RIPK3*
SKCM	*NLRC4, ZBP1, TNF, CASP1, AIM2, CASP5, MEFV, DFNA5, NLRP3, FADD, CASP3, ADAR,GSDMD, CASP8**	*CASP2, CASP3, CASP4*	*CASP10, CASP7, PYCARD, CASP4, CASP12, NLRP1, RIPK3, MLKL, TNFRSF1A*

	Primary Markers				Secondary Markers		
	(a) ∩ (b) ∩ (c)	(a) ∩ (b) – (a) ∩ (b) ∩ (c)	(a) ∩ (c) – (a) (b) (c)	(a) (Only)	(b) ∩ (c) – (a) ∩ (b) ∩ (c)	(b) (Only)	(c) (Only)
LGG	*CASP4, GSDMD, NLRC4, TNF, CASP7, DFNA5, FADD*	*CASP1, ZBP1, CASP5, CASP3, PYCARD, RIPK3, CASP8, TNFRSF1A, NLRP3, CASP6, MEFV, RIPK1*	*MLKL, CASP10*		*AIM2, CASP12, ADAR*	*NLRP1, CASP2*	
KIRC	*AIM2, NLRC4, NLRP3, CASP1, PYCARD*	*ZBP1, CASP5, MEFV, DFNA5, CASP4*	*TNF, RIPK3*		*CASP8, CASP3, CASP2, ADAR, RIPK1, CASP7, FADD*	*TNFRSF1A, CASP10, MLKL, NLRP9*	*CASP12*
SKCM	*ZBP1, TNF, CASP1, MEFV, RIPK3, NLRC4, NLRP1, DFNA5, TNFRSF1A*	*AIM2, CASP5, NLRP3, GSDMD*	*MLKL, CASP10, PYCARD, CASP4, CASP7*	*CASP8*	*CASP12, ADAR, FADD, CASP3*		*CASP2*

Supplementary Table 2: Cancer-specific bifurcation of PANoptosis markers.

LGG		TCGA (Train)	TCGA (CV)	GSE16011 (Test)	
Multivariate (Coxnet)	Primary	0.809	NA	0.608	
	Primary + Secondary	0.826	NA	0.615	
GLM Coxnet (GLMnet)	Primary	0.784	0.786	0.662	
	Primary + Secondary	0.819	0.801	0.649	
RFS	Primary	0.872	0.771	0.649	
	Primary + Secondary	0.924	0.781	0.662	
**KIRC**		**TCGA (Train)**	**TCGA (CV)**	**NG2699 (Test)**	
Multivariate (Coxnet)	Primary	0.675	NA	0.711	
	Primary + Secondary	0.715	NA	0.710	
GLM Coxnet (GLMnet)	Primary	0.670	0.670	0.655	
	Primary + Secondary	0.712	0.690	0.688	
RFS	Primary	0.834	0.645	0.669	
	Primary + Secondary	0.923	0.679	0.642	
**SKCM**		**TCGA (Train)**	**TCGA (CV)**	**GSE65904 (Test)**	**GSE22155 (Test)**
Multivariate (Coxnet)	Primary	0.688	NA	0.596	0.617
	Primary + Secondary	0.690	NA	0.594	0.618
GLM Coxnet (GLMnet)	Primary	0.663	0.673	0.613	0.588
	Primary + Secondary	0.681	0.670	0.610	0.587
RFS	Primary	0.767	0.612	0.602	0.613
	Primary + Secondary	0.794	0.626	0.602	0.662

Supplementary Table 3: Comparison of prognostic predictive capability of the different survival models on independent test sets for LGG, KIRC and SKCM using Harrell's CI as a quality metric.

LGG		TCGA (Train)	TCGA (CV)	GSE16011 (Test)	
Coxnet	Top	0.767	NA	0.696	
GLMNet	Top	0.760	0.794	0.615	
RFS	Top	0.865	0.774	0.672	
**KIRC**		**TCGA (Train)**	**TCGA (CV)**	**NG2699 (Test)**	
Coxnet	Top	0.672	NA	0.620	
GLMNet	Top	0.674	0.663	0.617	
RFS	Top	0.789	0.666	0.634	
**SKCM**		**TCGA (Train)**	**TCGA (CV)**	**GSE65904 (Test)**	**GSE22155 (Test)**
Coxnet	Top	0.672	NA	0.614	0.585
GLMNet	Top	0.672	0.664	0.614	0.585
RFS	Top	0.740	0.615	0.592	0.589

Supplementary Table 4: Comparison of different survival models built using only the key 'Top' PANoptosis markers on independent test sets for LGG, KIRC and SKCM using Harrell's CI as a quality metric.
